# Nanocarrier-mediated antioxidant delivery for liver diseases

**DOI:** 10.7150/thno.38834

**Published:** 2020-01-01

**Authors:** Senlin Li, Huiru Li, Xiaoding Xu, Phei Er Saw, Lei Zhang

**Affiliations:** 1 Guangdong Provincial Key Laboratory of Malignant Tumor Epigenetics and Gene Regulation, Sun Yat-sen Memorial Hospital, Sun Yat-sen University, Guangzhou 510120, People's Republic of China;; 2 Department of Hepatobiliary Surgery, Sun Yat-sen Memorial Hospital, Sun Yat-sen University, Guangzhou 510120, People's Republic of China.

**Keywords:** Liver disease, ROS, antioxidant, nanocarrier.

## Abstract

Liver is the principal detoxifying organ and metabolizes various compounds that produce free radicals (FR) constantly. To maintain the oxidative/antioxidative balance in the liver, antioxidants would scavenge FR by preventing tissue damage through FR formation, scavenging, or by enhancing their decomposition. The disruption of this balance therefore leads to oxidative stress and in turn leads to the onset of various diseases. Supplying the liver with exogeneous antioxidants is an effective way to recreate the oxidative/antioxidative balance in the liver homeostasis. Nevertheless, due to the short half-life and instability of antioxidants in circulation, the methodology for delivering antioxidants to the liver needs to be improved. Nanocarrier mediated delivery of antioxidants proved to be an ingenious way to safely and efficiently deliver a high payload of antioxidants into the liver for circumventing liver diseases. The objective of this review is to provide an overview of the role of reactive oxygen species (oxidant) and ROS scavengers (antioxidant) in liver diseases. Subsequently, current nanocarrier mediated antioxidant delivery methods for liver diseases are discussed.

## Reactive Oxygen Species (ROS)

### Formation and decomposition of ROS

When the body is exposed to exogenous or endogenous harmful irritants, oxidative stress occurs. Related redox metabolites free radicals, mainly reactive oxygen species (ROS) and reactive nitrogen species (RNS) will be produced. The intracellular ROS are primarily originated from the mitochondrial electron transport chain. Through complex I (NADH dehydrogenase), II and III (ubiquinone cytochrome C reductase) of the mitochondrial inner membrane 2, NADH can transfer electrons and hydrogen atoms to O_2_ to produce the non-toxic water product (H_2_O). However, when O_2_ accepts only one electron, it is reduced to superoxide (O_2_^-^ ), which is converted by superoxide dismutase (SOD) into hydrogen peroxide (H_2_O_2_). At high ROS levels, H_2_O_2_ can be catalyzed to hydroxyl radicals (·OH) by Fenton reaction. In addition, O_2_^-^ reacts with nitric oxide (NO) to form peroxynitrite (ONOO^-^) 3.

ROS can be classified into two groups depending on their electrons: radicals and non-radicals. ROS with unpaired electrons are also known as free radicals (FRs) and these include Superoxide (O_2_^•-^), Hydroxyl (OH^•-^),Alkoxyl radical (RO^•-^), and Peroxyl Radical (ROO^•-^)^4^. Due to the unpaired electrons, ROS has higher reactivity as it can either donate an electron or obtain one from other compounds to achieve stability. Therefore, the attacked molecule loses its electron and becomes a free radical, building a chain reaction cascade which finally damages the cell 5.

**Figure 1:**
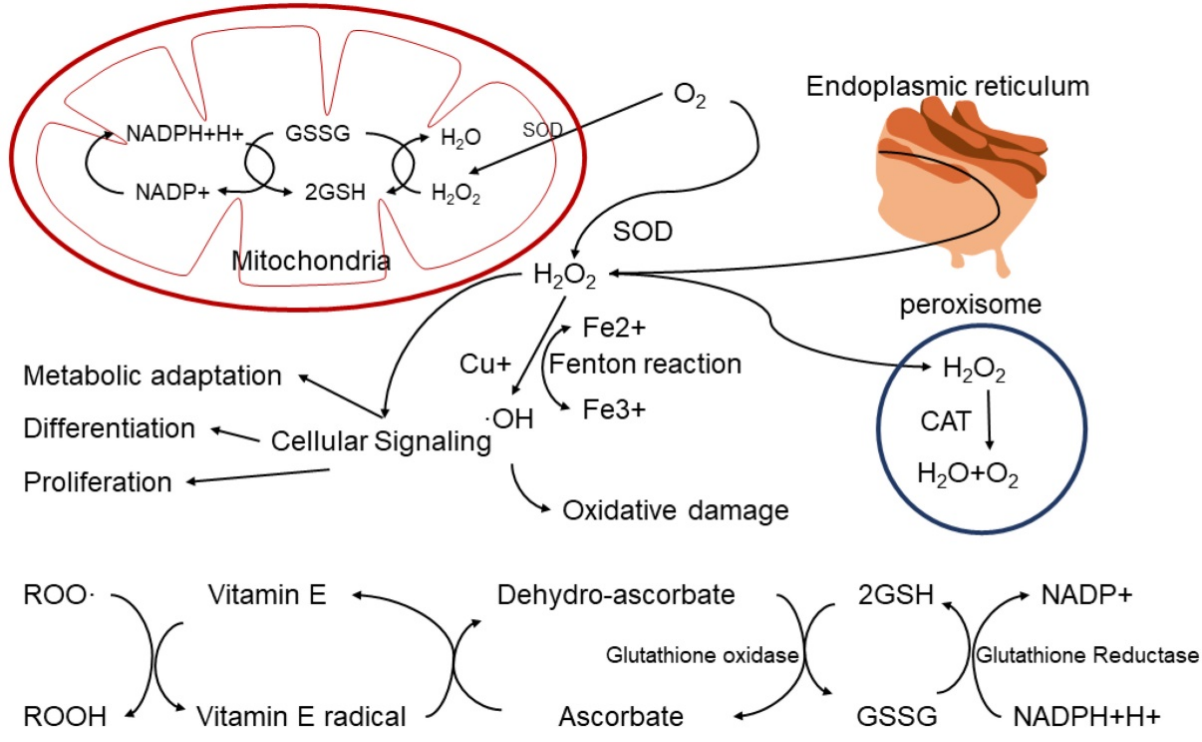
ROS production and consumption paths in biological system.

Non-radicals are oxidants which already have paired electrons, and these include Hydrogen peroxide (H_2_O_2_), Singlet oxygen (^1^O_2_), Ozone (O_3_), Organic peroxide (ROOH), Hypochlorous acid (HOCl), and Hypobromous acid (HOBr)^6^. These non-radical species also can easily lead to free radical reactions in living organisms 7. For example, Hydrogen peroxide(H_2_O_2_) is formed in a dismutation reaction catalyzed by the enzyme superoxide dismutase (SOD) *in vivo*. Although it is not a free radical, H_2_O_2_ could cause DNA damage to the cell at relatively low concentration (10 μM). This is due to fact that H_2_O_2_ can produce hydroxyl radical (OH^-^) in the presence of transition metal ions, which can cause DNA damage 8. However, H_2_O_2_ can also be decomposed into H_2_O and O_2_ in peroxisome, which is involved in multiple signal pathways in cells, including metabolic adaptation, cell differentiation and proliferation (Figure 1).

### Endogenous and exogenous ROS

**Figure 2:**
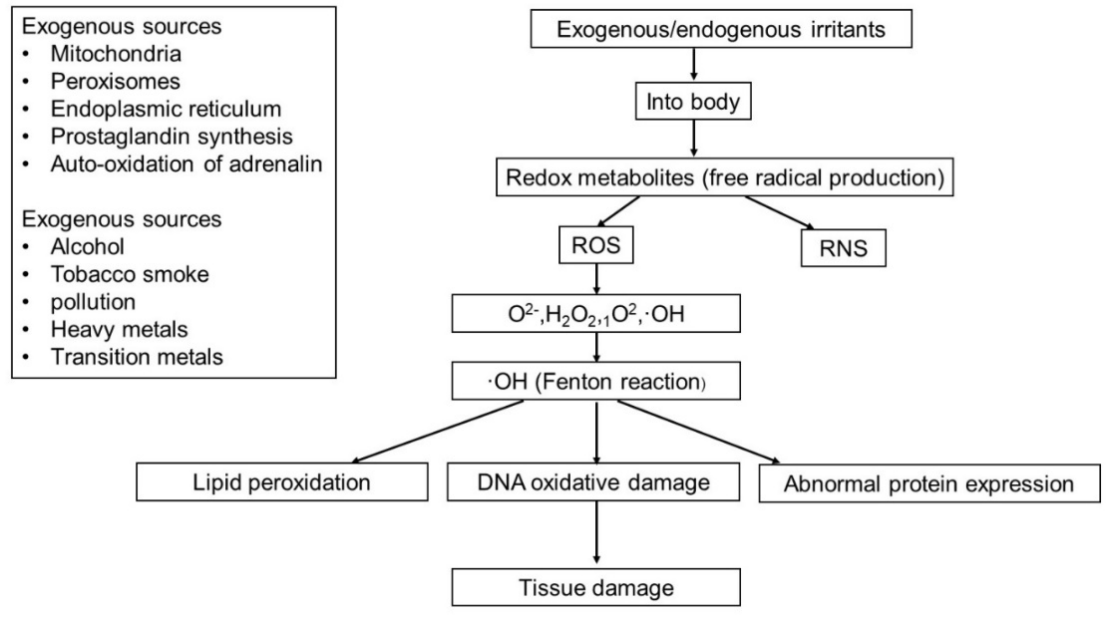
The sources of endogenous and exogenous ROS and the pathways of tissue damage caused by ROS.

ROS can be produced from either endogenous or exogenous sources. Endogenous sources of ROS include mitochondria, peroxisomes and endoplasmic reticulum (ER), where oxygen consumption is high. Other endogenous sources of ROS include prostaglandin synthesis, auto-oxidation of adrenaline, phagocytic cells, reduced riboflavin, reduced flavin mononucleotide (FMNH_2_), reduced flavin adenine dinucleotide (FADH_2_), cytochrome P450, immune cell activation, inflammation, mental stress, excessive exercise, infection, cancer, aging, and ischemia etc.^9^. ROS generated from exogenous sources includes pollution, alcohol, tobacco smoke, heavy metals, transition metals, industrial solvents, pesticides, certain drugs (i.e. halothane and paracetamol) and radiation (Figure 2).

Low levels of ROS/RNS have been proven to have beneficial effects and are involved in various physiological functions such as in immune function (i.e. defense against pathogenic microorganisms), cellular signaling pathways, mitogenic response and redox regulation 10. By increasing the reactivity of cells, excess ROS can cause DNA oxidative damage 11, abnormal protein expression 12 and lipid peroxidation 13, as well as the inactivation of certain enzymes and cofactors (Figure 2). Eventually, the accumulation of various abnormal substances leads to diseases, such as cancer, diabetes, cardiovascular disease, and nervous system diseases 14.

### ROS/redox balance in liver

**Figure 3:**
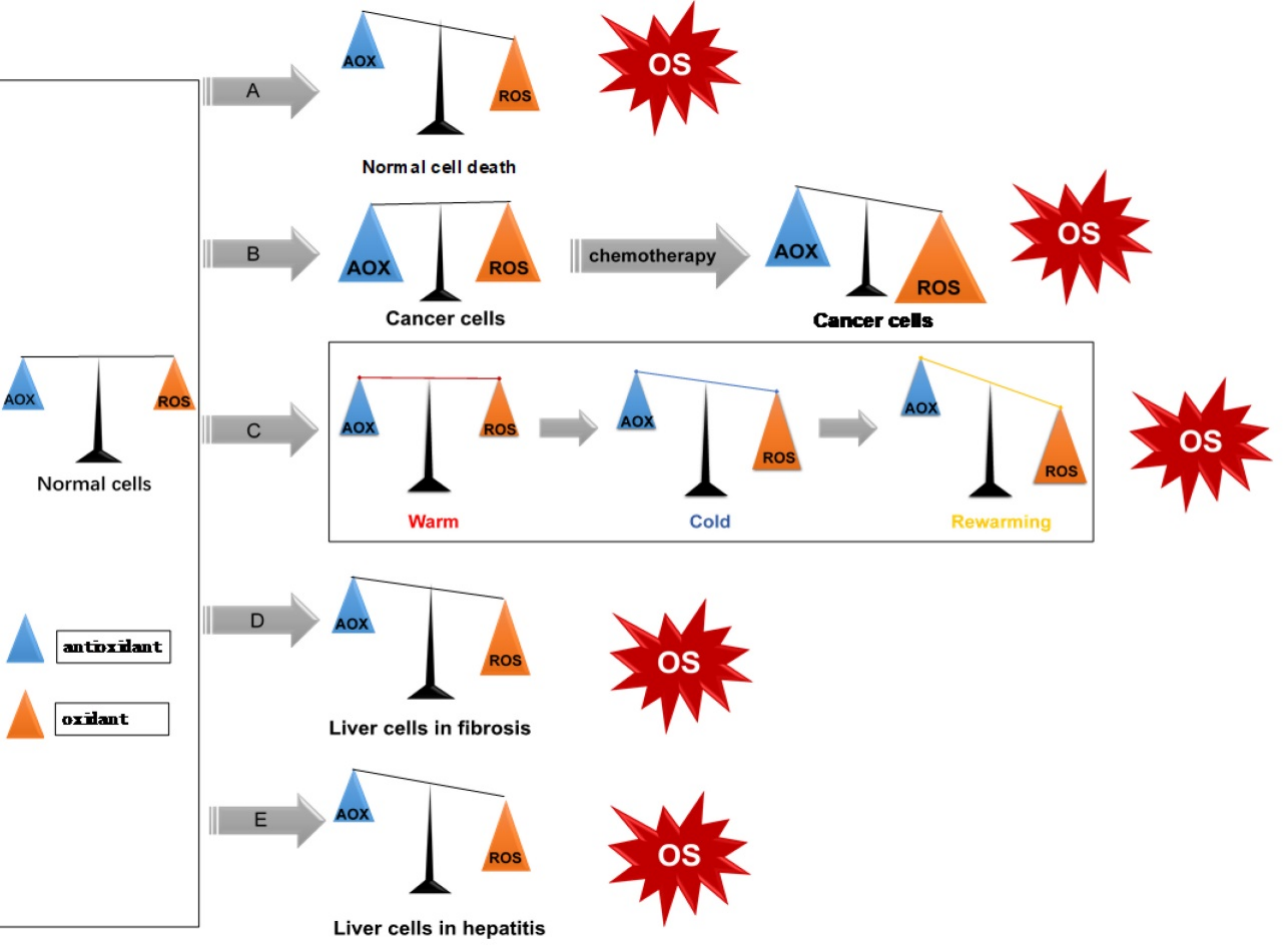
ROS/redox imbalance in liver diseases. (**A**) Apoptosis, or programmed cell death; (**B**) Liver tumor cells have higher oxidation/antioxidant levels, after use of chemotherapy drugs, induces cell death by increasing ROS levels; (**C**) ROS in liver transplantation. Upon reperfusion, KCs are activated and dramatically release oxygen free radicals, which leads to liver damage; (**D-E**): HBV and HCV induce chronic hepatitis and liver fibrosis through enhancing oxidative stress response.

ROS exist ubiquitously, and together with the ROS clearance system participate in intracellular redox balance (i.e. homeostasis). When oxidative stress occurs, excessive production of ROS and/or consumption of antioxidants increases, and the redox homeostasis becomes imbalanced, indicating the onset or occurrence of a disease (Figure 3). In normal conditions, cells have low levels of ROS which act as intracellular signaling molecules 15. They play important roles in maintaining various biochemical reactions, such as signal transduction, inflammatory response, and autophagy 16.

### ROS in liver diseases

#### ROS in viral hepatitis

Hepatitis B virus (HBV) is a double-stranded DNA virus which can selectively infect hepatic cells and persists epically. Hepatitis C virus (HCV) is a single-stranded RNA virus that encodes a single polyprotein of about 3000 amino acids. Both HBV and HCV can induce chronic hepatitis and liver fibrosis through enhancing oxidative stress response, which induces DNA damage 17. The accumulation of DNA damage causes liver cancer 18. When HBV/HCV infects normal liver cells, the cells become necrotic with injury and inflammation phenotype. At the same time, the human immune system attempts to eliminate them, which increases oxidative stress in liver cells as inflamed immune cells generate excessive ROS and RNS 19. Accumulated data strongly suggests that ROS/RNS can damage normal cell mismatch repair function and that persistent chronic inflammation caused by hepatitis virus can increase the mutation load of normal cells, which triggers specific oncogenic pathways and finally cause a malignant mutation process in normal cells. HBV infection can activate the Kupffer cells (KCs) to produce various proinflammatory cytokines, such as Interleukin-1 (IL-1), Interleukin-6 (IL-6), C-X-C Motif Chemokine Ligand 8 (CXCL-8, known as IL-8) and tumor necrosis factor-α (TNF-α)^20^. These abnormally produced cytokines can destroy mitochondrial cytochrome oxidase (complex IV), block the electron transport chain, promote ROS levels and induce Hepatocellular Carcinoma (HCC). Chronic HBV infection can also increase the total amount of iron (Fe) in the liver. The accumulated ferric iron (Fe^3+^) in the liver is reduced to ferrous iron (Fe^2+^). Excessive Fe^2+^ in the liver is highly toxic and is one of the major influencing factors of liver cancer. The HBV genome can encode various gene products, including HBV-encoded X protein (HBx), which could potentially cause liver cancer. However, exactly how HBx causes normal cell carcinogenesis has not been fully elucidated. The expression of HBx protein in para-cancerous tissues was slightly higher than in cancer tissues, but the difference was not significant (*p* > 0.05). This indicates that HBx protein itself may not directly participate in the development of liver cancer 21. Ha et al. found that HBx-induced ROS activates hepatocellular carcinogenesis via dysregulation of the phosphate gene 22. Wang et al. found that HBx can induce active oxygen production in normal liver cell line LO2 through the nuclear factor kappa-B (NF‐κB) signaling pathway, which could partially clarify how HBV causes HCC 23.

HCV infection activates antigen-presenting cells (APCs), KCs, and dendritic cells (DCs) in the liver and triggers persistent inflammation that causes continuous apoptosis and regeneration of liver cells 24. During this cycle, high turn-over of hepatocytes leads to a high occurrence of DNA mutations which in turn damage the hepatocytes' normal function and progresses to HCC 25. One study found that HCV-associated HCC patients had higher oxidative stress marker 8-hydroxy-2' -deoxy guanosine (8-OHdG) and reactive oxygen metabolites than HBV-related HCC patients, indicating more oxidative stress from HCV infection 26. Furthermore, serological tests also indicated that the iron accumulation in HCV-infected hepatocytes (especially in lysosomes) was always elevated.

#### ROS in liver fibrosis and cirrhosis

Hepatic stellate cells (HSC) and KCs are associated with the occurrence and development of cirrhosis 27, 28. Activated HSC can transform into myofibroblast cells (MFCs), which are involved in the formation of liver fibrosis and the reconstruction of intrahepatic structures by proliferating and secreting extracellular matrix. *In vivo*, ROS and O^2-^ activate HSC, induce collagen production and induce damage to hepatocytes 29. ROS triggers the activation of NF-κB, which increases cell survival by inhibiting apoptosis. NF-κB also regulates several genes involved in cell transformation, proliferation, and angiogenesis. The activation of NF-kB increases nitrogen monoxide (NO) and ROS production. NO and ROS are involved in the formation of oxidized low-density lipoprotein (OxLDL) which further activate NF-κB and forms a vicious cycle that causes hepatocyte damage^30^. In liver fibrosis and cirrhosis condition, KCs are constantly activated, therefore resulting in the production of large amounts of ROS, which in turn induces extracellular ROS and causes hepatocyte necrosis 31.

#### ROS in liver cancer (HCC)

The carcinogenicity of ROS is primarily attributed to the genotoxicity of ROS in diverse cellular processes 32. Gene mutations caused by ROS are associated with DNA base modifications. When the base of a key oncogene or tumor suppressor gene is modified, it may cause tumor occurrence and development. For example, the majority of mutations induced by ROS in the P53 tumor suppressor gene transfer guanine (G) to thymine (T) 33. Free radicals cause different types of chemical changes in DNA, which could be mutagenic 34. ROS can damage DNA by inducing base modifications, deletions, strand breakage, chromosomal rearrangements and hyper- and hypo-methylation of DNA 35. Cancer cells in particular, in comparison to normal cells, have higher levels of ROS and are more susceptible to mitochondrial dysfunction due to their higher metabolic rate 36. Cancer cells display elevated levels of oxidative stress due to activation of oncogenes and loss of tumor suppressors 37. By altering growth signals and gene expression, ROS cause continuous proliferation of cancer cells 38.

Some studies elucidated the importance between the extracellular redox state and cancer cell aggressiveness. The extracellular ROS/RNS can damage the ECM and plasma membranes, which may change their overall structure and the regulation of cellular adhesion, proliferation, migration, and cell signaling. Extracellular redox participates in proliferation, adhesion/migration, invasion and survival of cancer cells in both blood circulation and lymph node 39.

#### ROS in liver transplantation

Liver transplantation is the ultimate treatment for patients with end-stage liver disease. However, during the process of transplantation, ischemic injury-reperfusion (I/R) of the graft can lead to organ dysfunction or primary nonfunction 40. Hepatic I/R injury occur when there is a short blockage of blood supply to the liver and subsequent re-establishment of the blood supply. The severity of the damage depends on the duration of ischemia, but the injury process is more extensive during the reperfusion period than the ischemia 41. The injury is characterized by the production of ROS, disturbance of the microcirculation, and activation of the coagulation system.

The I/R injury is classified into three types: warm ischemic, cold ischemic and rewarming, depending on whether the ischemic organ is located *in situ* (warm), or undergoing cold ischemia preservation (cold)^42^. Rewarming ischemia typically occurs during transplantation process of the graft, when the cold liver is subjected to room or body temperature while performing the vascular reconstruction, also termed reperfusion 43. The I/R injury mainly damages the sinusoidal endothelial cell (SEC). Platelets induce SEC apoptosis on reperfusion of the cold ischemic liver 44. NO production by platelets in combination with ROS synthesis on reoxygenation can lead to the formation of reactive nitrogen species (RNS), which is a highly reactive inducer of apoptosis in endothelial cells 45. KCs are activated upon reperfusion; and become the main source of vascular ROS 46 which leads to an increased phagocytosis, lysosomal enzymes, and various cytokines including tumor necrosis factor α (TNF-α)^47^.

Furthermore, during the early stage after reperfusion (< 2 hours), the dramatic increase of oxygen free radicals leads to liver cell death 48. The late phase of liver injury (6 - 48 hours) is an inflammatory disorder thought to be mediated by recruited neutrophils. Neutrophils release proteolytic enzymes and ROS, which contribute to the damage of hepatocytes and sinusoidal endothelial cells (SEC).The early and late stages together comprise the development of hepatic I/R injury 49. It has been determined that both necrosis (during the extended ischemic phase) and apoptosis (during the late phase of reperfusion) occur in hepatic I/R injury; the entire I/R procedure is an oncotic process 50.

Liver I/R injury is not only related to the reactive oxygen species (ROS)-generating system, but also to xanthine/xanthine oxidase (XOD) 51. During ischemia, xanthine dehydrogenase (XDH), the physiologic form other enzyme, is converted to the oxygen radical-producing form XOD 52. Concurrently, there is an accumulation of xanthine, the substrate for XOD. On reoxygenation, XOD reacts with molecular oxygen to produce ROS. In fact, the mitochondrion also sustains injury and becomes a significant source of ROS 53. In isolated hepatocytes subjected to anoxia and reoxygenation, mitochondria were identified as sources of ROS formation that caused cell injury 54. Then, the free radical scavenging system in ischemic tissue is impaired, which aggravates the damage of free radicals to the tissue after ischemic reperfusion. The main determinant of reperfusion injury is ischemic time. If the time of ischemia was short, there was no obvious reperfusion injury after reperfusion.

### ROS in acute liver injury caused by sepsis

**Figure 4:**
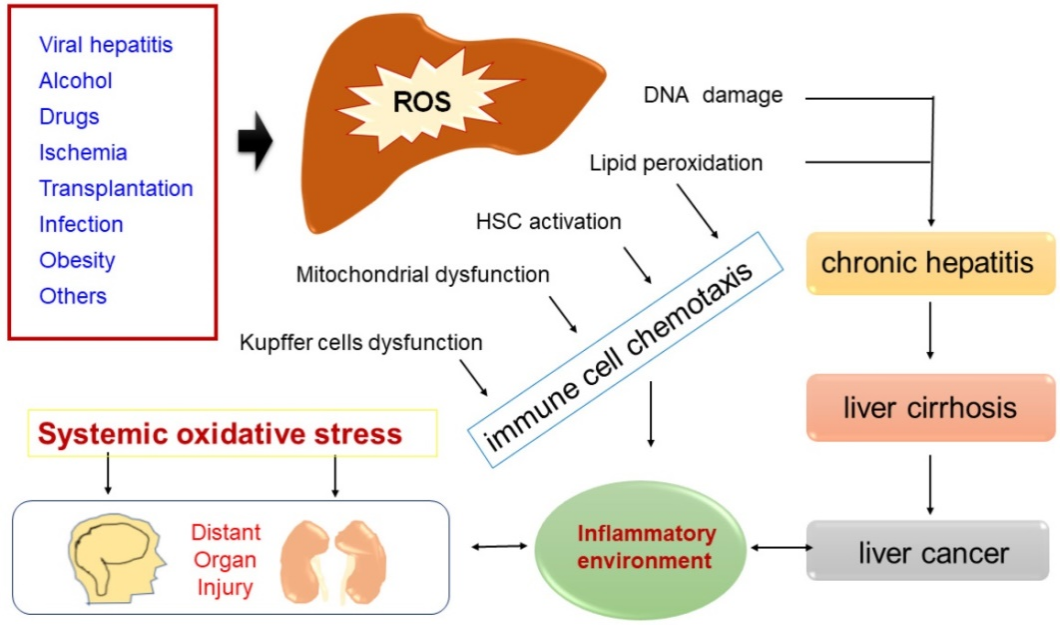
Common causes of ROS accumulation that lead to DNA damage, lipid peroxidation, HSC activation, mitochondrial dysfunction or Kupffer cells dysfunction. Increased ROS-mediated DNA damage and lipid peroxidation cause chronic hepatitis, some chronic hepatitis goes to cirrhosis, and some cirrhosis gradually evolves into liver cancer. In some disease states (such as sepsis/hepatic failure), a sharp rise in ROS in the liver can cause systemic immune activation, which in turn damages distant organs.

Acute liver injury caused by multiple factors can easily evolve into sepsis when combined with bacterial infection. Sepsis is an uncontrolled response of a host to an external infection characterized by the release of large amounts of pro-inflammatory and anti-inflammatory cytokines. Patients with cirrhosis have increased risk to develop sepsis, sepsis-induced organ failure, and sepsis-related death. Microbes express macromolecular motifs, named microorganism-associated molecular patterns (MAMPs), which are recognized by the immune system via receptors called pathogen recognition receptors (PRRs) 55. Pathogen recognition receptors mediate the activation of immune cells and the synthesis and release of inflammatory cytokines, which help to eliminate pathogen infections within a controlled range. But when combined with cirrhosis, biliary tract infections, etc., the above-mentioned anti-infective process is more likely to be out of control, manifested as a markedly imbalanced cytokine response, which converts responses that are normally beneficial for fighting infections into excessively damaging inflammation (**Figure 4**). Tissue hypoperfusion and hypoxia play an important role in the development of organ failure. In order to reduce perfusion pressure and blood flow, the formation of microthrombus reduces the deformability of red blood cells and tissue edema caused by increased capillary permeability of blood vessels due to imbalance before thrombus. In addition, cells may not be able to properly utilize existing oxygen due to impaired mitochondrial respiratory function, in part due to excess production of ROS and RON. Cell infiltration, especially neutrophils, directly damages tissues by releasing lysosomal enzymes and superoxide radicals. In addition, tissue damage and cell damage release components that are recognized by the immune cells as alarm factors, maintaining the inflammatory process as a vicious circle 56.

## Antioxidant

### Types of antioxidants

Antioxidant treatments include increasing endogenous antioxidants, external supplementation of exogenous antioxidants, and strategies to reduce oxidative stress, such as disrupting the ROS-producing electron transport chain. ROS clearance systems include antioxidant enzymes and non-enzymatic ROS scavengers. Common antioxidant enzymes are: superoxide dismutase (SOD), catalase and family members of peroxidase. Catalase catalyzes H_2_O_2_ into H_2_O and O_2_, and ONOO^-^ decomposed by catalase and glutathione peroxidase. Glutathione (GSH) and NAD(P)H are common non-enzymatic ROS scavengers, which are capable of delivering electrons to O_2_. GSH is oxidized to glutathione disulfide (GSSG), which is reduced to GSH by glutathione reductase (GSR). Nuclear factor erythroid 2-related factor 2(Nrf2), which is a major regulator of cellular antioxidants, activates the antioxidant response element (ARE) and activates downstream genes encoding antioxidant proteins such as GSR. Nrf2 also regulates other antioxidant pathways such as GSH and NADPH 57.

### Antioxidants in the treatment of liver diseases

Antioxidants have the effect of scavenging ROS, reducing intracellular ROS levels and preventing oxidative damage. Curcumin, Resveratrol, Ebselen (glutathione peroxidase analog), and Vitamin E, among others, have been reported to have antioxidant effects, and can be used as exogenous antioxidants to enter tumor cells to reduce ROS. Proper intake of antioxidants has been shown to reduce the risk of cancer and delay cancer progression 58.

One major source of ROS during HCV infection is NAD(P)H oxidase (Nox) proteins which consist of Nox1, 2, 3, 4, 5 and Duox1 and 2 59. Hepatocytes and Huh7 human hepatoma cells have been found to express Nox family enzymes *in vitro* 60. A study also indicated that hepatocyte Nox1 and Nox4 are prominent sources of ROS during complete HCV replication 61. Diphenylene iodonium (DPI), which was used to decrease ROS generation by HCV core protein by Okuda et al., is also an inhibitor of flavoproteins and commonly used to inhibit Nox 62.

In the treatment of liver fibrogenesis, Pyrroquinoline-quinone has been demonstrated to suppress oxidative stress in mice 63. This natural compound with antioxidant and anti-inflammatory properties has been proved to have hepatoprotective effect in the livers of rats with secondary biliary cirrhosis 64. Mitochondrial reduced glutathione (mGSH) plays an important role in the therapeutic potential of superoxide scavengers, and the combined approach of these natural agents with mGSH replenishment may be important in steatohepatitis and liver fibrosis 65.

Curcumin can eliminate lipid radicals in the cell membrane and become a phenoxy radical, so it is considered as a very potent lipid-soluble antioxidant 66. Curcumin exhibited chelating activity and is able to capture ferrous ion through its functional carbonyl groups 66. Curcumin can degrade under basic pH after 30 min into ferulic acid and vanillin which are also themselves antioxidants 67. Curcumin was also capable of decreasing oxidative stress by alterations of glutathione levels 68. Curcumin has been examined in hepatic fibrosis induced by carbon tetrachloride (CCl_4_) in rats. The experiment indicated that treatment with curcumin reduced serum and tissue cholesterol profiles and hepatic enzyme 119. Bruck et al. showed that curcumin inhibited thioacetamide-induced cirrhosis in rats 68.

Recent studies showed pomegranate fruit and flower extracts to exhibit free-radical scavenging properties, playing a role in hepatoprotection and prevention of liver injury 69. Some studies found that Pomegranate emulsion (PE) is able to counter dietary carcinogen diethyl nitrosamine (DENA)-induced rat hepatocarcinogenesis. After PE treatment (1 or 10 g/kg), striking chemo-preventive results were demonstrated by reduced incidence, number, multiplicity, size and volume of hepatic nodules, all precursors of HCC. PE also alleviated DENA-induced hepatic lipid peroxidation and protein oxidation, and elevated protein and messenger RNA expression of the Nrf2 70.

Silymarin is a polyphenolic antioxidant derived from silybinin, which has hepatoprotective properties and is very widely used in chronic hepatitis and cirrhosis as well as toxic liver damage 71.Silymarin is a mixture of flavonolignans, and is a very widely used in herbal medicine and as dietary supplement in the treatment of alcoholic liver disease, acute and chronic viral hepatitis, and toxigenic liver damage 72. Previous studies have indicated comparable effects for silymarin and silybinin on the antioxidative properties and growth inhibition of cancer cells 73, 74.

### Current Methods to reduce ROS

#### Ischemic preconditioning

Repeated, brief ischemic preconditioning training on the liver can stimulate the production and release of endogenous protective substances (such as antioxidants) to mitigate and resist the subsequent prolonged ischemic and hypoxic injuries. Fernandez et al. reported that preconditioning protects against both cold and warm hepatic ischemia-reperfusion injury by preventing xanthine oxidase-derived oxidant stress 75. Ischemic preconditioning reduced the conversion of xanthine dehydrogenase (XDH) to xanthine oxidase (XOD) and limited the accumulation of xanthine in liver grafts during cold ischemia 76.

#### Gene therapy

Under inflammation, trauma or other stressful conditions, the gene expression of most antioxidant enzymes, such as SOD, glutathione peroxidase, catalase or heme oxygenase-1 (HO-1) is a key mechanism for the body in response to various ROS. Adenoviral vectors of Cu/Zn-SOD, Mn-SOD, and EC-SOD have been shown to protect against warm ischemia reperfusion (I/R) injury in mouse livers 77. Ref-1 is known to act as a redox-dependent regulator of various transcription factors 78, Ozaki et al. reported that adenoviral overexpression of Ref-1 in hepatic tissue results in significant suppression of reperfusion-induced oxidative stress, NF-kB activation, apoptosis, and acute hepatic injury 79. Redox factor-1 introduced into liver grafts by adenoviral vectors not only protected them from ischemia/reperfusion-induced injury, but accelerated regeneration in a partial liver transplant model.

### The drawbacks of antioxidants

Previous clinical studies have not shown the efficacy of antioxidants to prevent cancer 80. While low concentrations of antioxidants do protect cells from oxidative damage, high levels actually induce ROS and cytotoxicity 81. For example, quercetin has both antioxidant and oxidative effects and the activity of GSH and GR (Glutathione Reductase) is decreased in oxidative stress cells. Long-term supplementation of quercetin does not increase GSH, but destroys the GSH metabolic pathway and promotes oxidation 82. Unstable GSH is difficult to use as a therapeutic drug but adding GR increases GSH stability and improve the effectiveness of scavenging ROS. In fact, the final ROS levels will increase in a NADPH-dependent and thioredoxin reductase dependent manner 83.

In addition, the failure of antioxidants may be that they do not effectively target mitochondria, which is the site of ROS production. Tumor cells are more susceptible to ROS than normal cells 84. Resveratrol and α-tocopherol succinate (vitamin E analogs) directly target mitochondria to exert anticancer effects, inducing a large number of ROS production which causes tumor cell death 85. Antioxidants promote tumor growth by reducing ROS-induced cancer cell death 86, 87. Excessive antioxidants also cause cytotoxic substances, such as excess Epigallocatechin-3-gallate (EGCG) or selenium 88, 89. Some studies have shown that inhibiting the production of antioxidants or inhibiting ROS clearance in cells can trigger cancer cell deaths 90, 91. The disadvantages of each method are summarized in Table 1 below.

### Combination therapy

#### Antioxidant + chemotherapy

Chemotherapy is a systemic infusion of drugs for the treatment of advanced cancer. However, due to severe side effects such as emesis, liver function lesion, and myelosuppression, there is no proven effective systemic chemotherapy for HCC. Since 2002, transcatheter arterial chemoembolization (TACE) has become the standard treatment for patients with stage B liver cancer, which has fewer systemic complications and side effects 92, 93. TACE can be divided into Hepatic arterial infusion chemotherapy (TAI) and Hepatic arterial embolization (TAE). The process of TAI includes: inserting a catheter from the femoral artery into the hepatic artery, and then dripping with chemotherapeutic drugs in order to have effect on local tumor tissues. The process of TAE includes: inserting a catheter into the tumor's blood supply artery, then injecting with an appropriate amount of embolic agent to occlude the target artery, and reach the tumor starvation effect. Chemotherapy medicine combined with iodide oil was used to embolize the tumor's peripheral blood vessels, and then a large amount of chemotherapy was injected. Finally, a gelatin sponge was used to embolize the proximal end of the blood supply artery. This method utilizes the hepatic artery as the source of drug administration. As the hepatic artery supplies almost 100% of the blood supply to liver tumors, TACE promises less systemic toxicity with higher drug retention in tumor 94, 95.

Standard chemotherapy drugs for TACE are mitomycin, gemcitabine, interferon, oxaliplatin, doxorubicin and fluorouracil 96. Although TACE is considered the primary therapy of liver cancer, it has many drawbacks such as hypohepatia, gastrointestinal reaction, fever, and other complications (acute liver failure, acute upper gastrointestinal bleeding, hepatapostema, pulmonary artery embolism, etc.) Chemotherapy lacks the ability to specifically kill tumor cells 97. Therefore, elevated ROS caused by chemotherapy is also the source of many side effects. On the one hand, chemotherapy combined with anti-oxidation treatment can reduce the side effects of chemotherapy to a certain extent; on the other hand, this may also weaken the tumor killing effect caused by chemotherapy. Therefore, although there are benefits in using the combination of antioxidant with chemotherapy, nevertheless the safety and efficacy of combination therapy with antioxidants and chemotherapy is controversial 98, 99.

In fact, some *in vitro* and *in vivo* studies have confirmed that large doses of food-borne antioxidants can induce tumor cell differentiation and apoptosis and inhibit its proliferation 100, 101. Haba et al. found that the antioxidant vitamin K2 can significantly reduce the probability of liver cirrhosis becoming liver cancer after viral hepatitis 101. To reduce severe liver dysfunction caused by chemoembolization, antioxidant glutathione is often used in clinical work.

Therefore, considering the dual role of antioxidants in tumor chemotherapy, we need to assess the patient's chemotherapy tolerance before surgery, pay close attention to the patient's vital signs during surgery, give positive supportive treatment after surgery, and choose the appropriate type of antioxidant (used in a combination of drugs when necessary), to reduce the side effects of chemotherapy, while enhancing or not weakening the killing effect of chemotherapy on tumors.

#### Antioxidant + radiotherapy

When the expression of superoxide dismutase (SOD) is increased, cancer cells will exhibit stronger radioactive resistance 106, and when SOD or the activity of GSH or NADH is lowered, the radiosensitivity of cancer cells is increased 107. Therefore, the combination of additional antioxidants could reduce ROS and remove ROS from cell damage, thereby protecting cancer cells from death 108.

In fact, anti-oxidation therapy combined with radiotherapy is often widely used as a means of reducing the side effects of radiotherapy. Pathak et al. found that the use of multiple antioxidant mixtures helps tumor patients' successfully complete radiotherapy with a prolonged median survival 109. Studies have shown that radiotherapy combined with high-dose ATO (vitamin E + vitamin C + β-carotene + vitamin A + coenzyme Q) can enhance the efficacy of radiotherapy and chemotherapy and reduce tumor recurrence 110.

It is worth mentioning that some antioxidants can synergistically work towards the tumor killing effect of radiotherapy and chemotherapy by inducing tumor cell apoptosis and cell cycle arrest mechanism 111. Quercetin is a potent cancer therapeutic agent and dietary antioxidant present in fruit and vegetables which prevents tumor proliferation by inducing cell cycle arrest. Quercetin nanoparticle accelerates the cleavage of caspase-9, caspase-3, and induces the up-releasing of cytochrome C, contributing to apoptosis in liver cancer cells. Quercetin nanoparticle effectively inhibits liver cancer cell proliferation, cell migration and colony formation, thus suppressing liver cancer progression. Carnosic acid (CA), as a phenolic diterpene with anticancer, antibacterial, antidiabetic, as well as neuroprotective properties, is produced by many species from Lamiaceae family. Administration of CA nanoparticles was sufficient to considerably inhibit liver cancer progression through inhibition of inflammation and acceleration of apoptosis in liver cancer by altering NF-κB activation and activating caspase-3 through the Bad pathway 112.

## Nanocarrier mediated delivery for liver diseases

Most drugs rely on delivery agents for safe and target-oriented transport *in vivo*, especially large biomacromolecules (i.e. RNA-based drugs and protein-based drugs)^113^, ^114^. Specific targeting of these drugs allows it to reach intracellular receptors or organelles, therefore achieving specific targeting and therapeutic effect. There are many types of delivery vehicles with sub-micro to nanometer scale, generally known as Nanoparticles (NPs): liposomes, polymer nanoparticles, microsomes (micelles), dendritic molecules, protein nanoparticles, viral NP, exosomes and natural membrane NP, metal and metal-derived NP, carbon nanomaterial and hybrid NP 115-117. NPs not only have the ability to encapsulate therapeutic agents and target and control release, but also improve the solubility of unmodified drug compounds in the circulation 118. NPs also have the advantage of large ratio of volume to surface area, shell variability, low biodegradability and low cytotoxicity 119. Liposome is the first approved nanomedicine (e.g., liposomal doxorubicin, LD); doxorubicin liposomal, Doxil)^120^. In 1995, Doxil was approved for clinical use in the treatment of AIDS-related Kaposi's sarcoma, ovarian cancer and other cancers 121. In 2016, NP albumin-binding paclitaxel (Nab paclitaxel; Abraxane) was the second nanomedicine to be approved. Both drugs improve biodistribution, cellular uptake and internalization of drugs by encapsulating drugs with NPs and targeting disease sites.

The development of treatment protocols for nanocarrier-mediated liver diseases has grown rapidly in recent years and has shown great application prospects 122, 123. Taking liver cancer as an example, tumor cells are significantly different from normal cells in gene and extrinsic phenotype, and the resulting microenvironment changes are used to efficiently target tumors and alleviate the side effects of systemic intravenous administration. Targeting strategies fall into two categories: passive targeting and active targeting. In the case of passive targeting, nanomedicines can reach tumors via the leaky vasculature of the tumors by the enhanced permeability and retention (EPR) effect^124^. Diversiform stimuli including pH, enzymatic, redox and light have been used for passive targeting of nanoparticles oncotherapy 125-127. In the case of active targeting, asialoglycoprotein receptor (ASGPR) a commonly found lectin receptor which are profoundly expressed in liver cancer cells. Natural ligands such as asialofeutin as well as synthetic ligands (galactosylated cholesterol, glycolipids, arabinogalactan (AG), lactosylated/galactosylated polymers) has achieved specific liver ASGPR targeting^128^. Multiple receptors including Integrin receptors 129, Transferrin Receptors (TfR)^130^, Epidermal Growth Factor Receptors (EGFR)^131^, Folate Receptors (FR)^132^ and Glycyrrhetinic Acid (GA) Receptors^133^ have been used as specific molecules for active targeting to kill tumor cells. Research has found that low-density lipoprotein-based nanoparticles can act as a transporter for unesterified DHA (LDL-DHA) and demonstrates selective cytotoxicity toward HCC cells. Injection of LDL-DHA into the hepatic artery of rats selectively deregulated redox reactions in tumor tissues by increasing levels of reactive oxygen species and lipid peroxidation, depleting and oxidizing glutathione and nicotinamide adenine dinucleotide phosphate 134.

Nanocarrier-mediated antioxidant delivery systems are more focused on inflammatory liver diseases such as drug-induced acute liver injury or sepsis-induced acute liver failure. Go et al. developed pathological stimulus-activatable nanoplatforms (ketalized maltodextrin nanoparticles; KMD), which are able to deliver therapeutic and imaging functions to the acidic conditions simultaneously, as may be found in the site of inflammation^135^. KMD was synthesized as a platform of the theragnostic nanoparticles by conjugating acid-cleavable hydrophobic moieties to maltodextrin through carbonate bonds, which could undergo acid-triggered hydrolytic degradation to generate carbon dioxide (CO) bubbles, amplifying the ultrasound signal for detecting drug metabolism and distribution. The study used a cell culture model and a mouse model of acute liver failure induced by acetaminophen (APAP) to comprehensively evaluate the potential of silymarin-loaded KMD (s-KMD) nanoparticles as ultrasound contrast agents and therapeutic agents. s-KMD nanoparticles show significant ultrasound enhancement in the apo-poisoned liver and significantly inhibit liver damage by inhibiting the expression of pro-inflammatory cytokines. In rats liver injury with ethanol and Methamphetamine (METH) model, gold nanoparticles (GNPs) can exert anti-inflammatory, anti-oxidant and anti-fibrosis by down-regulating the activity of Kupffer cells and hepatic stellate cells 136.

Excessive ROS production plays a key role in sepsis-mediated liver failure. ROS-responsive nanoparticles (NPs) formed may function as an effective drug delivery system for alleviating sepsis-induced liver injury by preferentially releasing drug molecules at the disease site^137^. Chen et al. synthesized the ROS-responsive nano-form (mPEG-b-PPS-NP) of via-self-assembly of di-block copolymers of poly(ethylene glycol (PEG) and poly(propylene sulfide) (PPS). They conducted experiments on the platform to deliver the anti-oxidation treatment molecule melatonin (Mel), which has poor pharmacokinetic properties and limited therapeutic effects. The nano-platform is effectively melatonin encapsulated by oil-in-water emulsification technology 137. Using a mouse acute liver injury induced by sepsis showed that Mel-loaded mPEG-b-PPS-NP is biocompatible in reducing oxidative stress, inflammatory response and subsequent liver damage 137.

### Advantages of nanocarrier mediated antioxidant therapy

#### Good solubility and hydrophilicity

Hydrophilic surface modifications prolong the half-life of NP and allow sufficient time to reach the tumor site. The nanoparticles enter the tumor matrix through the defected microvascular endothelium around the tumor, and are retained due to blockage of lymphatic vessel reflux. This basic principle of enhanced permeability and retention (EPR) effects to achieve passive targeting of nanoparticles to solid tumors. Because of the tight junctions in the normal endothelial cell gap, it prevents particles larger than 2 nm from passing. However, the tight junctions of the tumor vasculature and the basement membrane are disordered, allowing 10 to 500 nm NP to exude and accumulate in tumor matrix 138. The lymphatic system of the tumor is also damaged; further intercepting macromolecular particles and delaying their outflow 139. So, the EPR effect allows tumors to retain more polymer NPs, proteins, liposomes and micelles than other tissues 140.

#### Enhances the stability and prolonged circulation half-life of antioxidant

**Figure 5:**
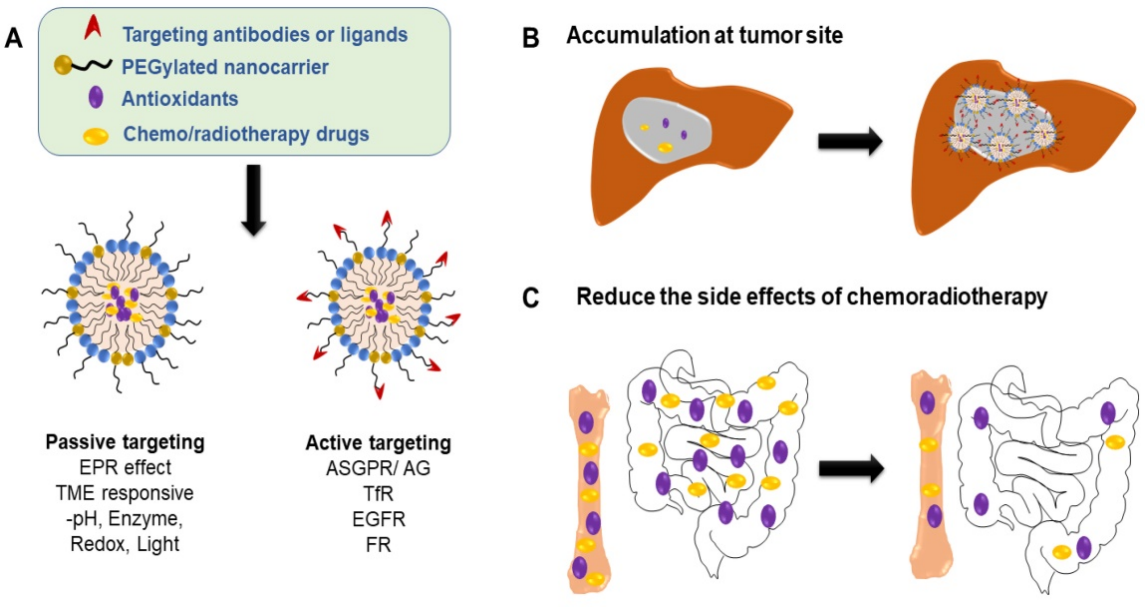
Application and strategy of nanocarrier-mediated antioxidant therapy in liver cancer: combined with radiotherapy and chemotherapy to reduce the side effects. (**A**) Nanocarriers can be designed to be passively or actively targeting tumor site. (**B**) Enrichment in tumor site increases tumor/organ ratio, thereby reducing the side effects of systemic intravenous chemotherapy (*e.g.,* gastrointestinal reactions and hematopoietic disorders, (**C**).

Most of the nanomedicines enter the body by intravenous injection. After entering the bloodstream, NP reacts non-specifically with serum protein, then the NP surface is covered with a hydrophobic "canopy"^119^. This "canopy" changes the physical properties of NP (such as particle size, stability and surface properties) and biological properties, affecting its pharmacokinetics *in vivo*, PK, biodistribution, cellular uptake and internalization, cytotoxicity, etc., thereby reducing the efficacy of nanomedicine 141. Moreover, the NP hydrophobic surface is rapidly recognized by the mononuclear phagocyte system (MPS) and cleared from circulation 142. Small molecules, although routinely used as antioxidant agents for cancer treatment, have characteristics that limit their use in clinical applications, including poor water solubility (discussed above) as well as non-specific removal by the vascular endothelial system and the mononuclear phagocytosis system. Due to their small size, these molecular substances were cleared mostly through liver and kidney metabolism, which is also the cause of toxic side effects, which in turn cause more damage to the liver and kidney. This is also the main reason why the biological half-life of small molecule drugs is short is not efficient therapeutically. 143. Nanomaterials are helpful to contain these small molecules, protecting drugs from early clearance, an acid-base environment, enzyme degradation. Along with surface modification, PEGylation and stimuli-responsiveness, nanocarrier is a solution to the problems mentioned above (Figure 5A).

#### PEGylation slows the clearance of Nano-antioxidant drugs in the circulation

Studies found that modification of the NP surface by polyethylene glycol (PEG) can protect it from MPS clearance, increasing circulating half-life and target cell uptake 138 PEGylation has become the gold standard for current NP coating^144^. For example, the PEGylated liposome Doxil is called "invisible liposome" and has a circulating half-life of up to 2 days 145. The polylactic acid (PLA)-PEG micelle form of paclitaxel also has longer half-life in circulation. It has been found that the protein species bound to the NP surface can be reduced and altered by increasing the PEG density of the NP surface, thereby reducing the phagocytosis of macrophages 146.

#### Active targeting of nanocarriers increases tumor specificity and reduces systemic toxicity of drug

In order to better target the site of the lesion and reduce the toxic side effects, researchers used molecular markers specifically expressed by tumor cells to develop specific targeted ligands or antibodies (Figure 5B-C), which were modified on the surface of the nanoparticles to enhance their ability to penetrate tumor tissue specifically targeted to target cells^129^, ^132^. PK2 (FCE28069), a HPMA-polymer-Gly-Phe-leu-Gly-doxorubicin conjugate that also contains the sugar galactosamine, was the first ligand-targeted nanoparticle to reach the clinic. The galactose-based ligand was used to target the ASGPR, which is high expressed in primary liver cancer cells. In a clinical study using PK2 to treat primary hepatocellular carcinoma, the concentrations of drug in the liver were 15-20% of the administered dose after 24 hours and the concentrations in the tumor were 12-50 folds higher than would have been achieved through free doxorubicin 147. Additionally, active targeted nanoparticles can be combined with intracellular directional release to further enhance the ability of nanodrugs to specifically target tumor tissue. For example, CAlAA-01 is a targeted nanoparticle that has proven multivalent binding to cancer cell surface, which has an active drug (siRNA) release mechanism that is triggered by pH decline below a value of 6.0 (which occurs in the endosomal pathway)^148^. Nanocarriers not only prolong the blood circulation of the drug, but also efficiently locate the tumor site through EPR effect, thus reducing the side effects of systemic drugs. For example, liposomal doxorubicin can significantly reduce the gastrointestinal reactions and myelosuppression caused by chemotherapy drugs 149.

#### Controlled drug release and increasing the bioavailability of antioxidants

NPs play an important role in nanomedicines such as Doxil^150^ and liposomal paclitaxel formulation (Genexol-PM)^151^ in increasing drug bioavailability, reducing cytotoxicity, and improving drug pharmacokinetics (PK). The principles that control the release of NP-encapsulated drugs mainly include pH or temperature changes and the enzymes-triggered drug release 152. As an example, our group synthesized a multifunctional envelope-type nanoparticle platform for prostate cancer (PCa)-specific *in vivo* siRNA delivery, which used sharp pH-responsive polymers (Meo-PEG-b-P(DPA-co-GMA) for self-assembly with siRNA. It not only significantly extended the blood cycle time but also used pH-triggered oligoarginine-mediated endosomal membrane penetration to release siRNA 153. Like chemotherapeutic drugs and siRNA, the proportion of exogenously injected antioxidants that actually reach the site of the lesion is less than 1% due to the efficient removal of foreign substances via liver, kidney and the mononuclear endothelial system. Nanocarrier-mediated therapy could promote long circulation of antioxidants while increasing bioavailability. In addition, poorly water-soluble antioxidant components could be chemically modified to form antioxidant-derived nanoparticles, which greatly increase bioavailability while still being able to encapsulate other materials in the core 154.

#### TME responsive nanoparticles can reduce tumor resistance and enhance the ability to target tumors

The tumor microenvironment (TME) describes the non-neoplastic cells and extracellular matrix (ECM) present in the tumor, which includes fibroblasts, blood vessels and the immune cells 155, 156. Solid tumors can have a significantly higher proportion of ECM proteins than non-cancerous tissue, resulting in decreased diffusivity of both medium and large molecular weight proteins through the interstitial space. The ECM, composed of various proteins (e.g. collagen, hyaluronic acid, proteoglycans), and the slightly positive charge of collagen may attract anionic nanoparticles and drugs, decreasing their availability to tumor cells 157. Therefore, in recent years, nanomaterials or nanoparticles with different stimuli response have been developed that can respond to environmental factors, such as pH, temperature, light, reduction/oxidation, and enzymes (Figure 5A). Stimuli-responsive nanoparticles can facilitate augmented drug release, efficient and uniform distribution of therapeutic drugs throughout the tumor and enhanced cellular uptake in response to the tumor microenvironment 158, 159. For example, poly(histidine) (pHis) is an attractive candidate that has been extensively used for the fabrication of a pH-sensitive drug delivery system. Poly (ethylene glycol) methyl ether acrylate-block poly(L-Lysine)-block-poly(L-Histidine) triblock co-polypeptides were synthesized for pH-responsive drug delivery. The nanoparticles were found to be stable at physiological pH (7.4) but were dramatically destabilized in acidic pH due to the presence of pHis blocks, which promote targeted release of the drug and enhance the ability to target tumors 160. By the way, using nanoparticles to normalize TME is also an effective treatment strategy that has great application prospects 161, 162.

## Nano-antioxidants

### Nanoparticle encapsulating-antioxidants (NP-antioxidant)

**Figure 6:**
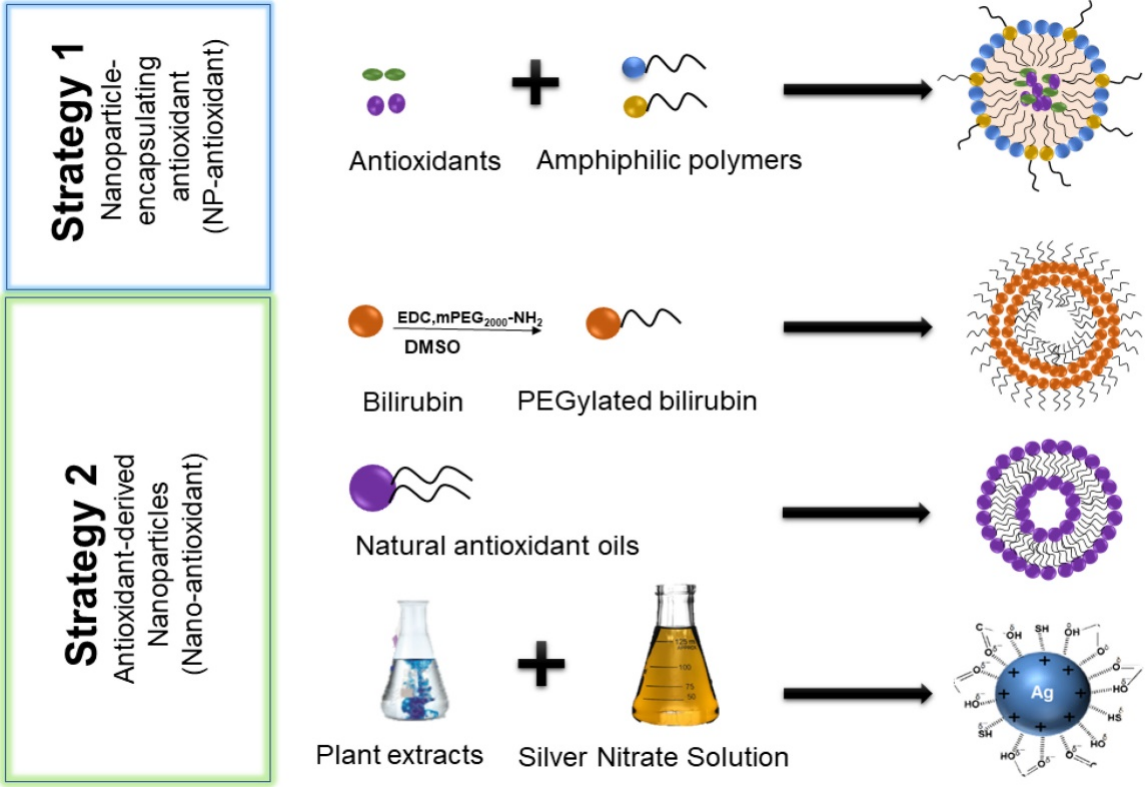
Two different strategies for the construction of antioxidant nano-drugs: Nanoparticle encapsulating-antioxidants (NP-antioxidant) and Antioxidant-derived nanoparticles (Nano-antioxidant).

Under physiological conditions, low levels of ROS maintain the body's normal metabolism and protect the body from bacterial viruses 16. An imbalance between ROS and antioxidants can cause a modification in signal pathways and carcinogenesis 163. Antioxidants can deplete ROS in tumor or tumor microenvironments as a means of treating tumors. However, problems such as short blood circulation time, poor membrane permeability, poor water solubility, and degradation of lysosome/endosomes seriously affect their efficacy 164. As mentioned above, nanocarrier systems could mitigate these shortcomings. Encapsulations of antioxidants into nanocarriers are termed Nano-antioxidants (**Figure 6**). Some examples of nanoparticle encapsulating antioxidants (NP-antioxidants) and antioxidant-derived nanoparticles (nano-antioxidants) are listed in **Table 2** below.

### Antioxidant-derived nanoparticles (nano-antioxidant)

The use of nanocarriers can improve the biological delivery of antioxidants and have documented therapeutic potential. In particular, the incorporation of antioxidants into nanoparticles can increase their bioavailability, improve targeting to the desired tissues or receptors, and provide controlled release of compounds over an extended period of time. However, because most reported nanocarriers are synthetic (i.e. polymers, lipids, inorganic metals, etc.) and do not possess intrinsic therapeutic efficacy, long-term application of these nanocarriers is associated with concerns regarding their degradation, elimination, and toxicity in humans 165. Furthermore, nanocarriers typically have low drug-loading capacities (typically below 10% for drugs), and this significantly reduces their accumulation in the tumor hence diminishing their therapeutic efficacy. Therefore, self-carrying Nano-antioxidant delivery systems that do not require the use of inner carriers could be seen as a paradigm shift in the therapeutic modality of liver diseases (Figure 5).

#### Natural oil-based lipid nanocarrier (NLC)

The natural oil-based lipid nanocarrier NLC published by Lacatusu et al. in 2015 is a hollow carrier and does not contain other drugs. Natural oils include grape seed oil, fish oil, and bay leaf oil, in which the essential oil of bay leaf has a scavenging effect on DPPH free radicals and inhibits the proliferation of K562 tumor cell lines 166. Anthocyanins in grape seed oil have been shown to induce cancer cell cycle arrest and apoptosis by activating DNA damage checkpoint cascades 167. Experiments show that the nanocarrier containing 25% grape seed oil and 2% bay leaf oil has the best ability to scavenge free oxygen radicals, and that the antioxidant activity value is 98%^168^.

#### *Agilen indicum* Silver Nanoparticles (AlAgNPs)

Plant extracts have been found to be useful in the synthesis of metal nanoparticles as they possess phyto-components like polyphenols, alkaloids, flavonoids, fatty acids and proteins which act as reducing and capping agents 169, 170. Silver nanoparticles (AgNPs) have high potential in cancer treatment. They cause selective disruption of the mitochondrial respiratory chain leading to high ROS which in turn cause DNA damage 171, 172. Biological synthesis of Abutilon indicum silver nanoparticles (AIAgNPs) published by Mata et al. in 2015, showed potent *in vitro* bioactivity, including free radical scavenging activity, antibacterial activity, and anticancer effect. The size of the nanoparticles is about 5-25 nm under transmission electron microscopy (TEM) which can kill tumor cells at lower concentrations (IC_50_= 3 µg/ml, 24h)^169^. There are also many medicinal plants and plant extracts that have been used for the synthesis of AgNPs e.g. *Azadirachta indica, Capsicum annuum, Magnolia kobus, Coriandrum sp.* etc. 173, 174. Therefore, biogenic AgNPs may become a potential cancer theragnostic agent in the near future 175.

#### Calcium phosphate nanoparticles (nano-CaP)

Calcium phosphate nanoparticles (nano-CaP) published by Mohammed et al. in 2016, showed great antioxidative and antitumor effects in the liver cancer model induced by diethylnitrosamine (DEN). DEN triggers the genotoxic effects of N-nitrosocarcinogens, inducing ROS production and DNA fragmentation 176. The activity of SOD and glutathione peroxidase in the liver was reduced, and the level of lipid peroxidation final product was increased, eventually causing oxidative damage of cells 177. Experiments showed that nano-CaP treatment blocks tumor proliferation by repairing fragmented DNA and promoting tumor cell apoptosis. It also increased the activity of liver alanine aminotransferase (ALT), OD and GPx, thereby reducing the damage of oxidative stress on cells, improving the oxidant/antioxidant balance and restoring liver function. On the other hand, gamma-glutamyl transferase(γ-GT), interleukin-2 (IL-2), interferon-γ(IFN-γ), tumor necrosis factor-α (TNF-α), metalloproteinase (MMP-9), heat shock protein-70 (HSP-70) and caspase-3 were also reduced 178.

#### PEGylated Bilirubin NPs (BRNPs)

Bilirubin is a powerful scavenger of various ROS and has the ability to modulate the immune system 179. Lee et al. first developed a water-soluble BRNP system that has a potent therapeutic effect for acute inflammatory diseases. By first conjugating PEG to bilirubin, (PEG-BR), these monomers then form self-assembled PEG-BR nanoparticles (BRNPs, 100 nm). In this study, a murine model of ulcerative colitis could be treated by intravenous BRNPs infusion and inhibited the progression of acute inflammation 180.

In a subsequent study, the authors examined the potential of BRNP to be used as a therapy for Ischemia-reperfusion injury (IRI). IRI is a major problem that may occur after liver transplantation, and leads to an increase in inflammation and apoptosis that result in the dysfunction of hepatic cells, organ rejection, and ultimately liver failure. It is likely that pro-inflammatory immune responses and increased oxidative stress damage the ischemic tissue after restoration of blood flow 181, 182. Herein, they found that the anti-oxidant properties of BRNPs protected primary hepatocytes from H_2_O_2_. In particular, mice pre-treated with BRNP prevented hepatocellular injury by reducing oxidative stress, production of pro-inflammatory cytokines, and neutrophil recruitment. Notably, these BRNPs preferentially accumulated in the lesions. These findings indicate that pretreatment with BRNP is a simple and safe approach to protect against IRI 154.

## Conclusion and future outlook

Maintaining a dynamic balance of ROS is the key to maintaining the health of the body. Increased ROS stress caused by a variety of factors, including exogenous alcohol, viruses, drugs, and endogenous insulin resistance, obesity, etc., are associated with a variety of liver diseases, including stratosis, hepatitis, liver fibrosis/cirrhosis and tumors, etc. 183, 184. In particular, chronic hepatitis-cirrhosis-hepatocarcinoma is called the trilogy of primary liver cancer evolutions, which owe to chronic inflammation and sustained ROS stimulation caused by hepatitis virus. Therefore, antioxidant therapy represents a reasonable strategy for prevention and treatment of liver disease due to the role of oxidative stress in initiation and progression of hepatic damage. In the field of liver cancer treatment, suitable antioxidants combined with radiotherapy and chemotherapy can not only enhance the tumoricidal effect of radiotherapy and chemotherapy, but also reduce side effects for patients. However, there is still a long way to go before antioxidant treatment enters the clinic. The complex mechanism of antioxidants in the physiological process, the lack of conclusive human research data and other difficulties hinder the clinical application of antioxidant therapy 183. Problems such as short blood circulation time, poor membrane permeability, poor water solubility, and degradation of lysosome/endosomes seriously affect its current clinical potential. The nanocarrier system can solve these problems well. Therefore, nanocarriers coated with antioxidants and nano-carriers constructed from natural antioxidants are used to enhance the efficacy of antioxidant treatments or to treat various diseases in combination with other drugs.

Although nanotechnology can solve many of the problems of antioxidant treatment, so far, most of the experiments have only stayed at the animal level. In animal experiments, nanomedicines are mostly administered by oral or intraperitoneal injection; this is also an influence for absorption and bio-availability of antioxidants. In addition, the liver acts as a central organ of metabolism and its functional status is closely related to other diseases, such as renal failure and abnormal glucose metabolism. Improvements in animal models are one of the more accurate assessments of treatment outcomes. For example, considering the important role of the liver in immune regulation, immune-sound mice are superior to immunodeficiency models such as nude mice. In addition, in tumor-related research, the humanized mouse or patient-derived tumor xenograft (PDX) model is closer to the true state of the human body, which helps us to comprehensively evaluate the efficacy of nano-antioxidant therapy. A number of variables still warrant thorough investigation: safe and effectives doses of antioxidants, treatment time, absorption, and bioavailability, given that there is a significant difference between humans and animals, and knowing that reactive oxygen species and oxidative stress play actives roles under certain conditions. In addition, the development of more effective nano-delivery systems and large-scale clinical research are beneficial to promote the development of antioxidant therapy.

## Figures and Tables

**Table 1:** The disadvantages of antioxidant therapy and chemotherapy / radiotherapy when administered alone.

Antioxidant therapy	Chemotherapy / radiotherapy
Only specific to one primary ROS, such as O_2_^·-^ or H_2_O_2_. No antioxidant is specific against secondary ROS/RNS.	Non-targeting, not cancer cell specific.
Non-targeting; which may induce increasing dose of drugs to reach the high concentration in target site, leads to more side effects.	TACE is invasive treatment with more complication, and do not significantly improve the survival rate, with high possibility of recurrence.
Unstable structure leads to short half-life, susceptibility to be converted into inactive form or cleared by RES or renal clearance.	Can damage the hepatic-renal function, even bring about other dysfunction and chronic pain, fever, gastrointestinal discomfort, etc.
Little effect if administered orally	Cancer acquire resistance to radiation and chemotherapy after several rounds of treatment

**Table 2:** NP-antioxidants and nano-antioxidants currently used in research and preclinical experiments.

Antioxidants	Source	Major activity	Reference	
Resveratrol (3,5,4'-trihydroxy-trans-stilbene)	Red grape skin, Japanese knotweed (*polygonum cuspidatum*), peanuts, blueberries and some other berries	**Anticarcinogenic activity:** Inhibits mammary carcinogenesis Anti-mutagenic and anticancer Induces human promyelocytic leukemia cell differentiation Inhibits cyclooxygenase and hydroperoxide functions Anti-proliferation and inhibit viability of human breasts epithelial cells* in vitro* Anti-initiation, anti-promotion, and anti-progression of tumors **Antioxidant:** Resveratrol-mediated inhibition was specific for the cyclooxygenase activity of COX-1 Inhibits reactive oxygen intermediates (ROI) generation and lipid peroxidation induced by tumor necrosis factor (TNF) Inhibits zymosan-induced oxygen radical production in murine macrophages and human monocytes and neutrophils Inhibits free-radical formation and cyclooxygenase activity **Anti-inflammatory:** Inhibits ROS and COX, and efficacy in skin and mammary animal models of tumorigenesis **Anti-thrombotic activities:** Selective estrogen receptor agonists and antagonists	185-188	
Genipin (GP)	Gardenia plant	**Anticarcinogenic activity:** Suppressing UCP2, intracellular pyruvic acid, and mitochondrial succinate dehydrogenase Chemo-preventive effect Cross-linking agent, drug delivery agent Natural availability, low cytotoxicity **Protective effect:** Hepatoprotective effect Ameliorate hepatic ischemia reperfusion injury, steatosis, autoimmune hepatitis, and fibrosis in rodents Ameliorates Galn/LPS induced hepatocellular damage by suppressing necroptosis-mediated inflammasome signaling	189-191	
Silymarin Silychristin	Silybinin	**Antioxidant activity:** Free radicals scavenging activity Cancer growth inhibition Lipid radical scavenger Anti-lipid oxidation, Anti-fibrosis Regulating Glutathione level	71, 73, 192, 193	
Pomegranate (PE)	Pomegranate fruit & flower	**Anticarcinogenic activity:** Anti-cancer activity (against DENA-induced rat hepatocarcinogenesis) **Antioxidant:** Free radicals scavenging activity Alleviate lipid peroxidation and protein oxidation	69, 70, 194, 195	
Pyrroloquinoline quinone	Some microorganism plant & animal tissues	**Antioxidant:** Oxidative stress suppression **Protective effect:** Hepatoprotective activity (anti-fibrosis)	63, 64	
Bilirubin	The final metabolite of the heme catabolic pathway	**Antioxidant:** Anti-inflammatory Scavenger of various ROS	154, 179, 180	

## Abbreviations

FR: free radicals
ROS: reactive oxygen species
RNS: reactive nitrogen species
SOD: superoxide dismutase
H_2_O_2_: hydrogen peroxide
ER: endoplasmic reticulum
FMNH_2_: reduced flavin mononucleotide
FADH_2_: reduced flavin adenine dinucleotide
HBV: hepatitis B virus
HCV: hepatitis C virus
KCs: Kupffer cells
IL-1: Interleukin-1
IL-6: Interleukin-6
CXCL-8: C-X-C Motif Chemokine Ligand 8
TNF-α: tumor necrosis factor-α
HBx: HBV-encoded X protein
APCs: antigen-presenting cells
DCs: dendritic cells
HCC: human hepatocellular carcinoma
HSC: Hepatic stellate cells
MFCs: myofibroblast cells
OxLDL: oxidized low-density lipoprotein
SEC: sinusoidal endothelial cell
I/R: ischemic injury-reperfusion
TNF-α: tumor necrosis factor-α
XOD: xanthine/xanthine oxidase
XDH: xanthine dehydrogenase
MAMPs: microorganism-associated molecular patterns
PRRs: pathogen recognition receptors
DPI: Diphenylene iodonium
PE: pomegranate emulsion
XOD: xanthine oxidase
TACE: transcatheter arterial chemoembolization
TAI: Hepatic arterial infusion chemotherapy
TAE: Hepatic arterial embolization
CA: Carnosic acid
LD: liposomal doxorubicin
Doxil: doxorubicin liposomal
EPR: enhanced permeability and retention
ASGPR: asialoglycoprotein receptor
TfR: Transferrin receptors
EGFR: Epidermal growth factor receptors
FR: Folate receptors
GA: Glycyrrhetinic Acid
GNPs: gold nanoparticles
γ-GT: gamma-glutamyl transferase
IL-2: interleukin-2
IFN-γ: interferon-γ
TNF-α: tumor necrosis factor-α
MMP-9: metalloproteinase-9
HSP-70: heat shock protein-70
PDX: patient-derived tumor xenograft

## References

[1] Halliwell B (1999). Oxygen and nitrogen are pro-carcinogens. Damage to DNA by reactive oxygen, chlorine and nitrogen species: measurement, mechanism and the effects of nutrition. Mutat Res.

[2] Brand MD (2010). The sites and topology of mitochondrial superoxide production. Exp Gerontol.

[3] Kumar S, Rhim WK, Lim DK, Nam JM (2013). Glutathione dimerization-based plasmonic nanoswitch for biodetection of reactive oxygen and nitrogen species. ACS Nano.

[4] Tabner BJ, Turnbull S, El-Agnaf O, Allsop D (2001). Production of reactive oxygen species from aggregating proteins implicated in Alzheimer's disease, Parkinson's disease and other neurodegenerative diseases. Curr Top Med Chem.

[5] Bayir H (2005). Reactive oxygen species. Crit Care Med.

[6] Kohen R, Nyska A (2002). Oxidation of biological systems: oxidative stress phenomena, antioxidants, redox reactions, and methods for their quantification. Toxicol Pathol.

[7] Genestra M (2007). Oxyl radicals, redox-sensitive signalling cascades and antioxidants. Cell Signal.

[8] Halliwell B, Clement MV, Long LH (2000). Hydrogen peroxide in the human body. Febs Letters.

[9] Cheeseman KH, Slater TF (1993). An introduction to free radical biochemistry. Br Med Bull.

[10] Nordberg J, Arner ES (2001). Reactive oxygen species, antioxidants, and the mammalian thioredoxin system. Free Radic Biol Med.

[11] Marnett LJ (2000). Oxyradicals and DNA damage. Carcinogenesis.

[12] Stadtman ER, Levine RL (2000). Protein oxidation. Ann N Y Acad Sci.

[13] Yla-Herttuala S (1999). Oxidized LDL and atherogenesis. Ann N Y Acad Sci.

[14] Schieber M, Chandel NS (2014). ROS function in redox signaling and oxidative stress. Curr Biol.

[15] Klein JA, Ackerman SL (2003). Oxidative stress, cell cycle, and neurodegeneration. J Clin Invest.

[16] Zhang J, Wang X, Vikash V, Ye Q, Wu D, Liu Y (2016). ROS and ROS-Mediated Cellular Signaling. Oxid Med Cell Longev.

[17] Georgakilas AG, Mosley WG, Georgakila S, Ziech D, Panayiotidis MI (2010). Viral-induced human carcinogenesis: an oxidative stress perspective. Mol Biosyst.

[18] Hagen TM, Huang S, Curnutte J, Fowler P, Martinez V, Wehr CM (1994). Extensive oxidative DNA damage in hepatocytes of transgenic mice with chronic active hepatitis destined to develop hepatocellular carcinoma. Proc Natl Acad Sci U S A.

[19] Murakami T, Kim T, Nakamura H (1998). Hepatitis, cirrhosis, and hepatoma. J Magn Reson Imaging.

[20] Hosel M, Quasdorff M, Wiegmann K, Webb D, Zedler U, Broxtermann M (2009). Not interferon, but interleukin-6 controls early gene expression in hepatitis B virus infection. Hepatology.

[21] Wang Q, Zhang T, Ye L, Wang W, Zhang X (2012). Analysis of hepatitis B virus X gene (HBx) mutants in tissues of patients suffered from hepatocellular carcinoma in China. Cancer Epidemiol.

[22] Ha HL, Yu DY (2010). HBx-induced reactive oxygen species activates hepatocellular carcinogenesis via dysregulation of PTEN/Akt pathway. World J Gastroenterol.

[23] Pandey MK, Sung B, Ahn KS, Kunnumakkara AB, Chaturvedi MM, Aggarwal BB (2013). Gambogic acid, a novel ligand for transferrin receptor, potentiates TNF-induced apoptosis through modulation of the nuclear factor-kappaB signaling pathway. Blood. 2007;110(10):3517-3525. Blood.

[24] Boltjes A, Movita D, Boonstra A, Woltman AM (2014). The role of Kupffer cells in hepatitis B and hepatitis C virus infections. J Hepatol.

[25] Arzumanyan A, Reis HM, Feitelson MA (2013). Pathogenic mechanisms in HBV- and HCV-associated hepatocellular carcinoma. Nat Rev Cancer.

[26] Nishimura M, Takaki A, Tamaki N, Maruyama T, Onishi H, Kobayashi S (2013). Serum oxidative-anti-oxidative stress balance is dysregulated in patients with hepatitis C virus-related hepatocellular carcinoma. Hepatol Res.

[27] Brandao DF, Ramalho LN, Ramalho FS, Zucoloto S, Martinelli Ade L, Silva Ode C (2006). Liver cirrhosis and hepatic stellate cells. Acta Cir Bras.

[28] Dixon LJ, Barnes M, Tang H, Pritchard MT, Nagy LE (2013). Kupffer cells in the liver. Compr Physiol.

[29] Bosch J, Abraldes JG, Fernandez M, Garcia-Pagan JC (2010). Hepatic endothelial dysfunction and abnormal angiogenesis: new targets in the treatment of portal hypertension. J Hepatol.

[30] Lin W, Tsai WL, Shao RX, Wu G, Peng LF, Barlow LL (2010). Hepatitis C virus regulates transforming growth factor beta1 production through the generation of reactive oxygen species in a nuclear factor kappaB-dependent manner. Gastroenterology.

[31] Kolios G, Valatas V, Kouroumalis E (2006). Role of Kupffer cells in the pathogenesis of liver disease. World J Gastroenterol.

[32] Poulsen HE, Prieme H, Loft S (1998). Role of oxidative DNA damage in cancer initiation and promotion. Eur J Cancer Prev.

[33] Hollstein M, Sidransky D, Vogelstein B, Harris CC (1991). p53 mutations in human cancers. Science.

[34] Dreher D, Junod AF (1996). Role of oxygen free radicals in cancer development. Eur J Cancer.

[35] Valko M, Izakovic M, Mazur M, Rhodes CJ, Telser J (2004). Role of oxygen radicals in DNA damage and cancer incidence. Mol Cell Biochem.

[36] Acuna UM, Wittwer J, Ayers S, Pearce CJ, Oberlies NH, EJ DEB (2012). Effects of (5Z)-7-oxozeaenol on the oxidative pathway of cancer cells. Anticancer Res.

[37] Cairns RA, Harris I, McCracken S, Mak TW (2011). Cancer cell metabolism. Cold Spring Harb Symp Quant Biol.

[38] Valko M, Leibfritz D, Moncol J, Cronin MT, Mazur M, Telser J (2007). Free radicals and antioxidants in normal physiological functions and human disease. Int J Biochem Cell Biol.

[39] Chaiswing L, Oberley TD (2010). Extracellular/microenvironmental redox state. Antioxid Redox Signal.

[40] Kupiec-Weglinski JW, Busuttil RW (2005). Ischemia and reperfusion injury in liver transplantation. Transplant Proc.

[41] Selzner N, Rudiger H, Graf R, Clavien PA (2003). Protective strategies against ischemic injury of the liver. Gastroenterology.

[42] Clavien PA, Harvey PR, Strasberg SM (1992). Preservation and reperfusion injuries in liver allografts. An overview and synthesis of current studies. Transplantation.

[43] Jaeschke H, Lemasters JJ (2003). Apoptosis versus oncotic necrosis in hepatic ischemia/reperfusion injury. Gastroenterology.

[44] Sindram D, Porte RJ, Hoffman MR, Bentley RC, Clavien PA (2000). Platelets induce sinusoidal endothelial cell apoptosis upon reperfusion of the cold ischemic rat liver. Gastroenterology.

[45] Gow AJ, Thom SR, Ischiropoulos H (1998). Nitric oxide and peroxynitrite-mediated pulmonary cell death. Am J Physiol.

[46] Caldwell-Kenkel JC, Currin RT, Tanaka Y, Thurman RG, Lemasters JJ (1991). Kupffer cell activation and endothelial cell damage after storage of rat livers: effects of reperfusion. Hepatology.

[47] Rudiger HA, Clavien PA (2002). Tumor necrosis factor alpha, but not Fas, mediates hepatocellular apoptosis in the murine ischemic liver. Gastroenterology.

[48] He SQ, Zhang YH, Venugopal SK, Dicus CW, Perez RV, Ramsamooj R (2006). Delivery of antioxidative enzyme genes protects against ischemia/reperfusion-induced liver injury in mice. Liver Transpl.

[49] Glantzounis GK, Salacinski HJ, Yang W, Davidson BR, Seifalian AM (2005). The contemporary role of antioxidant therapy in attenuating liver ischemia-reperfusion injury: a review. Liver Transpl.

[50] Teoh NC, Farrell GC (2003). Hepatic ischemia reperfusion injury: pathogenic mechanisms and basis for hepatoprotection. J Gastroenterol Hepatol.

[51] Muller MJ, Vollmar B, Friedl HP, Menger MD (1996). Xanthine oxidase and superoxide radicals in portal triad crossclamping-induced microvascular reperfusion injury of the liver. Free Radic Biol Med.

[52] Engerson TD, McKelvey TG, Rhyne DB, Boggio EB, Snyder SJ, Jones HP (1987). Conversion of xanthine dehydrogenase to oxidase in ischemic rat tissues. J Clin Invest.

[53] Arai M, Thurman RG, Lemasters JJ (2000). Contribution of adenosine A(2) receptors and cyclic adenosine monophosphate to protective ischemic preconditioning of sinusoidal endothelial cells against Storage/Reperfusion injury in rat livers. Hepatology.

[54] Yin DP, Sankary HN, Chong AS, Ma LL, Shen J, Foster P (1998). Protective effect of ischemic preconditioning on liver preservation-reperfusion injury in rats. Transplantation.

[55] Gustot T, Durand F, Lebrec D, Vincent JL, Moreau R (2009). Severe sepsis in cirrhosis. Hepatology.

[56] Abraham E, Singer M (2007). Mechanisms of sepsis-induced organ dysfunction. Crit Care Med.

[57] Gorrini C, Harris IS, Mak TW (2013). Modulation of oxidative stress as an anticancer strategy. Nat Rev Drug Discov.

[58] Cabello CM, Bair WB 3rd, Wondrak GT (2007). Experimental therapeutics: targeting the redox Achilles heel of cancer. Curr Opin Investig Drugs.

[59] Lambeth JD, Kawahara T, Diebold B (2007). Regulation of Nox and Duox enzymatic activity and expression. Free Radic Biol Med.

[60] Reinehr R, Becker S, Keitel V, Eberle A, Grether-Beck S, Haussinger D (2005). Bile salt-induced apoptosis involves NADPH oxidase isoform activation. Gastroenterology.

[61] de Mochel NS, Seronello S, Wang SH, Ito C, Zheng JX, Liang TJ (2010). Hepatocyte NAD(P)H oxidases as an endogenous source of reactive oxygen species during hepatitis C virus infection. Hepatology.

[62] Okuda M, Li K, Beard MR, Showalter LA, Scholle F, Lemon SM (2002). Mitochondrial injury, oxidative stress, and antioxidant gene expression are induced by hepatitis C virus core protein. Gastroenterology.

[63] Jia D, Duan F, Peng P, Sun L, Ruan Y, Gu J (2015). Pyrroloquinoline-quinone suppresses liver fibrogenesis in mice. PLoS One.

[64] Serviddio G, Bellanti F, Stanca E, Lunetti P, Blonda M, Tamborra R (2014). Silybin exerts antioxidant effects and induces mitochondrial biogenesis in liver of rat with secondary biliary cirrhosis. Free Radic Biol Med.

[65] von Montfort C, Matias N, Fernandez A, Fucho R, Conde de la Rosa L, Martinez-Chantar ML (2012). Mitochondrial GSH determines the toxic or therapeutic potential of superoxide scavenging in steatohepatitis. J Hepatol.

[66] Ak T, Gulcin I (2008). Antioxidant and radical scavenging properties of curcumin. Chem Biol Interact.

[67] Sharma RA, Gescher AJ, Steward WP (2005). Curcumin: the story so far. Eur J Cancer.

[68] Reyes-Gordillo K, Segovia J, Shibayama M, Tsutsumi V, Vergara P, Moreno MG (2008). Curcumin prevents and reverses cirrhosis induced by bile duct obstruction or CCl4 in rats: role of TGF-beta modulation and oxidative stress. Fundam Clin Pharmacol.

[69] Celik I, Temur A, Isik I (2009). Hepatoprotective role and antioxidant capacity of pomegranate (Punica granatum) flowers infusion against trichloroacetic acid-exposed in rats. Food Chem Toxicol.

[70] Bishayee A, Bhatia D, Thoppil RJ, Darvesh AS, Nevo E, Lansky EP (2011). Pomegranate-mediated chemoprevention of experimental hepatocarcinogenesis involves Nrf2-regulated antioxidant mechanisms. Carcinogenesis.

[71] Sersen F, Vencel T, Annus J (2006). Silymarin and its components scavenge phenylglyoxylic ketyl radicals. Fitoterapia.

[72] Saller R, Meier R, Brignoli R (2001). The use of silymarin in the treatment of liver diseases. Drugs.

[73] Gazak R, Walterova D, Kren V (2007). Silybin and silymarin-new and emerging applications in medicine. Curr Med Chem.

[74] Bhatia N, Zhao J, Wolf DM, Agarwal R (1999). Inhibition of human carcinoma cell growth and DNA synthesis by silibinin, an active constituent of milk thistle: comparison with silymarin. Cancer Lett.

[75] Fernandez L, Heredia N, Grande L, Gomez G, Rimola A, Marco A (2002). Preconditioning protects liver and lung damage in rat liver transplantation: role of xanthine/xanthine oxidase. Hepatology.

[76] Green CJ, Healing G, Simpkin S, Gower J, Fuller BJ (1989). Allopurinol inhibits lipid peroxidation in warm ischaemic and reperfused rabbit kidneys. Free Radic Res Commun.

[77] Wheeler MD, Katuna M, Smutney OM, Froh M, Dikalova A, Mason RP (2001). Comparison of the effect of adenoviral delivery of three superoxide dismutase genes against hepatic ischemia-reperfusion injury. Hum Gene Ther.

[78] Yan M, Xu W, Lu L, Sun L, Liu X, Zheng Z (2000). Induction of ref-1 ensures AP-1 activation in intracellular oxidative environment of IL-2-stimulated BA/F3beta cells. Biochem Biophys Res Commun.

[79] Ozaki M, Suzuki S, Irani K (2002). Redox factor-1/APE suppresses oxidative stress by inhibiting the rac1 GTPase. FASEB J.

[80] Burr M, Appleby P, Key T, Thorogood M (2001). Plasma ascorbic acid and risk of heart disease and cancer. Lancet.

[81] Poljsak B, Milisav I (2018). The Role of Antioxidants in Cancer, Friends or Foes?. Curr Pharm Des.

[82] Cialdella-Kam L, Nieman DC, Sha W, Meaney MP, Knab AM, Shanely RA (2013). Dose-response to 3 months of quercetin-containing supplements on metabolite and quercetin conjugate profile in adults. Br J Nutr.

[83] Szende B, Tyihak E, Kiraly-Veghely Z (2000). Dose-dependent effect of resveratrol on proliferation and apoptosis in endothelial and tumor cell cultures. Exp Mol Med.

[84] Gogvadze V, Orrenius S, Zhivotovsky B (2008). Mitochondria in cancer cells: what is so special about them?. Trends Cell Biol.

[85] Fulda S, Galluzzi L, Kroemer G (2010). Targeting mitochondria for cancer therapy. Nat Rev Drug Discov.

[86] Block KI (2004). Antioxidants and cancer therapy: furthering the debate. Integr Cancer Ther.

[87] Zhang Q, Zhang F, Li S, Liu R, Jin T, Dou Y (2019). A Multifunctional Nanotherapy for Targeted Treatment of Colon Cancer by Simultaneously Regulating Tumor Microenvironment. Theranostics.

[88] Galati G, Lin A, Sultan AM, O'Brien PJ (2006). Cellular and *in vivo* hepatotoxicity caused by green tea phenolic acids and catechins. Free Radic Biol Med.

[89] Lipinski B (2005). Rationale for the treatment of cancer with sodium selenite. Med Hypotheses.

[90] Trachootham D, Zhou Y, Zhang H, Demizu Y, Chen Z, Pelicano H (2006). Selective killing of oncogenically transformed cells through a ROS-mediated mechanism by beta-phenylethyl isothiocyanate. Cancer Cell.

[91] Raj L, Ide T, Gurkar AU, Foley M, Schenone M, Li X (2011). Selective killing of cancer cells by a small molecule targeting the stress response to ROS. Nature.

[92] Ishikawa T, Abe S, Watanabe T, Nozawa Y, Sano T, Iwanaga A (2016). Improved survival with double platinum therapy transcatheter arterial infusion using cisplatin and transcatheter arterial chemoembolization using miriplatin for BCLC-B hepatocellular carcinoma. Mol Clin Oncol.

[93] Amisaki M, Honjo S, Morimoto M, Hanaki T, Arai Y, Tokuyasu N (2016). The Negative Effect of Preoperative Transcatheter Arterial Chemoembolization on Long-Term Outcomes for Resectable Hepatocellular Carcinoma: A Propensity Score Matching Analysis. Yonago Acta Med.

[94] Cabrera R, Nelson DR (2010). Review article: the management of hepatocellular carcinoma. Aliment Pharmacol Ther.

[95] Ortega Lopez N (2015). PET/Computed Tomography in Evaluation of Transarterial Chemoembolization. PET Clin.

[96] Albert M, Kiefer MV, Sun W, Haller D, Fraker DL, Tuite CM (2011). Chemoembolization of colorectal liver metastases with cisplatin, doxorubicin, mitomycin C, ethiodol, and polyvinyl alcohol. Cancer.

[97] Zhou Z, Song J, Tian R, Yang Z, Yu G, Lin L (2017). Activatable Singlet Oxygen Generation from Lipid Hydroperoxide Nanoparticles for Cancer Therapy. Angew Chem Int Ed Engl.

[98] Lawenda BD, Kelly KM, Ladas EJ, Sagar SM, Vickers A, Blumberg JB (2008). Should supplemental antioxidant administration be avoided during chemotherapy and radiation therapy?. J Natl Cancer Inst.

[99] Cheng T, Liu J, Ren J, Huang F, Ou H, Ding Y (2016). Green Tea Catechin-Based Complex Micelles Combined with Doxorubicin to Overcome Cardiotoxicity and Multidrug Resistance. Theranostics.

[100] Taper HS, Jamison JM, Gilloteaux J, Summers JL, Calderon PB (2004). Inhibition of the development of metastases by dietary vitamin C:K3 combination. Life Sci.

[101] Habu D, Shiomi S, Tamori A, Takeda T, Tanaka T, Kubo S (2004). Role of vitamin K2 in the development of hepatocellular carcinoma in women with viral cirrhosis of the liver. JAMA.

[102] Klement RJ, Abbasi-Senger N, Adebahr S, Alheid H, Allgaeuer M, Becker G (2019). The impact of local control on overall survival after stereotactic body radiotherapy for liver and lung metastases from colorectal cancer: a combined analysis of 388 patients with 500 metastases. BMC Cancer.

[103] Carrier F, Liao Y, Mendenhall N, Guerrieri P, Todor D, Ahmad A (2019). Three Discipline Collaborative Radiation Therapy (3DCRT) Special Debate: I would treat prostate cancer with proton therapy. J Appl Clin Med Phys.

[104] Hilgard P, Muller S, Hamami M, Sauerwein WS, Haberkorn U, Gerken G (2009). [Selective internal radiotherapy (radioembolization) and radiation therapy for HCC-current status and perspectives]. Z Gastroenterol.

[105] Tsai CL, Hsu FM, Cheng JC (2016). How to Improve Therapeutic Ratio in Radiotherapy of HCC. Liver Cancer.

[106] Wang H, Jiang H, Van De Gucht M, De Ridder M (2019). Hypoxic Radioresistance: Can ROS Be the Key to Overcome It?.

[107] Jayakumar S, Kunwar A, Sandur SK, Pandey BN, Chaubey RC (2014). Differential response of DU145 and PC3 prostate cancer cells to ionizing radiation: role of reactive oxygen species, GSH and Nrf2 in radiosensitivity. Biochim Biophys Acta.

[108] Borek C (2004). Antioxidants and radiation therapy. J Nutr.

[109] Pathak AK, Bhutani M, Guleria R, Bal S, Mohan A, Mohanti BK (2005). Chemotherapy alone vs. chemotherapy plus high dose multiple antioxidants in patients with advanced non small cell lung cancer. J Am Coll Nutr.

[110] Biesalski HK, Frank J (2003). Antioxidants in cancer therapy: is there a rationale to recommend antioxidants during cancer therapy?. Biofactors.

[111] Ren KW, Li YH, Wu G, Ren JZ, Lu HB, Li ZM (2017). Quercetin nanoparticles display antitumor activity via proliferation inhibition and apoptosis induction in liver cancer cells. Int J Oncol.

[112] Tang B, Tang F, Wang Z, Qi G, Liang X, Li B (2016). Upregulation of Akt/NF-kappaB-regulated inflammation and Akt/Bad-related apoptosis signaling pathway involved in hepatic carcinoma process: suppression by carnosic acid nanoparticle. Int J Nanomedicine.

[113] Paulo CS, Pires das Neves R, Ferreira LS (2011). Nanoparticles for intracellular-targeted drug delivery. Nanotechnology.

[114] Lamprecht A, Benoit JP (2006). Etoposide nanocarriers suppress glioma cell growth by intracellular drug delivery and simultaneous P-glycoprotein inhibition. J Control Release.

[115] Zhang C, Ling CL, Pang L, Wang Q, Liu JX, Wang BS (2017). Direct Macromolecular Drug Delivery to Cerebral Ischemia Area using Neutrophil-Mediated Nanoparticles. Theranostics.

[116] Sharma A, Liaw K, Sharma R, Zhang Z, Kannan S, Kannan RM (2018). Targeting Mitochondrial Dysfunction and Oxidative Stress in Activated Microglia using Dendrimer-Based Therapeutics. Theranostics.

[117] Kim GW, Kang C, Oh YB, Ko MH, Seo JH, Lee D (2017). Ultrasonographic Imaging and Anti-inflammatory Therapy of Muscle and Tendon Injuries Using Polymer Nanoparticles. Theranostics.

[118] Chrastina A, Massey KA, Schnitzer JE (2011). Overcoming *in vivo* barriers to targeted nanodelivery. Wiley Interdiscip Rev Nanomed Nanobiotechnol.

[119] Davis ME, Chen ZG, Shin DM (2008). Nanoparticle therapeutics: an emerging treatment modality for cancer. Nat Rev Drug Discov.

[120] Chang HI, Yeh MK (2012). Clinical development of liposome-based drugs: formulation, characterization, and therapeutic efficacy. Int J Nanomedicine.

[121] Barenholz Y (2012). Doxil(R)-the first FDA-approved nano-drug: lessons learned. J Control Release.

[122] Baig B, Halim SA, Farrukh A, Greish Y, Amin A (2019). Current status of nanomaterial-based treatment for hepatocellular carcinoma. Biomed Pharmacother.

[123] Dai Y, Yang Z, Cheng S, Wang Z, Zhang R, Zhu G (2018). Toxic Reactive Oxygen Species Enhanced Synergistic Combination Therapy by Self-Assembled Metal-Phenolic Network Nanoparticles.

[124] Varshosaz J, Farzan M (2015). Nanoparticles for targeted delivery of therapeutics and small interfering RNAs in hepatocellular carcinoma. World J Gastroenterol.

[125] Chen F, Zhang J, Wang L, Wang Y, Chen M (2015). Tumor pH(e)-triggered charge-reversal and redox-responsive nanoparticles for docetaxel delivery in hepatocellular carcinoma treatment. Nanoscale.

[126] Chen Y, Liu X, Yuan H, Yang Z, von Roemeling CA, Qie Y (2019). Therapeutic Remodeling of the Tumor Microenvironment Enhances Nanoparticle Delivery. Adv Sci (Weinh).

[127] Huang WC, Chen SH, Chiang WH, Huang CW, Lo CL, Chern CS (2016). Tumor Microenvironment-Responsive Nanoparticle Delivery of Chemotherapy for Enhanced Selective Cellular Uptake and Transportation within Tumor. Biomacromolecules.

[128] Hu J, Liu J, Yang D, Lu M, Yin J (2014). Physiological roles of asialoglycoprotein receptors (ASGPRs) variants and recent advances in hepatic-targeted delivery of therapeutic molecules via ASGPRs. Protein Pept Lett.

[129] Chen L, Liu Y, Wang W, Liu K (2015). Effect of integrin receptor-targeted liposomal paclitaxel for hepatocellular carcinoma targeting and therapy. Oncol Lett.

[130] Martin DN, Uprichard SL (2013). Identification of transferrin receptor 1 as a hepatitis C virus entry factor. Proc Natl Acad Sci U S A.

[131] Liu Y, Wu X, Gao Y, Zhang J, Zhang D, Gu S (2016). Aptamer-functionalized peptide H3CR5C as a novel nanovehicle for codelivery of fasudil and miRNA-195 targeting hepatocellular carcinoma. Int J Nanomedicine.

[132] Liu MC, Liu L, Wang XR, Shuai WP, Hu Y, Han M (2016). Folate receptor-targeted liposomes loaded with a diacid metabolite of norcantharidin enhance antitumor potency for H22 hepatocellular carcinoma both *in vitro* and *in vivo*. Int J Nanomedicine.

[133] Cai Y, Xu Y, Chan HF, Fang X, He C, Chen M (2016). Glycyrrhetinic Acid Mediated Drug Delivery Carriers for Hepatocellular Carcinoma Therapy. Mol Pharm.

[134] Wen X, Reynolds L, Mulik RS, Kim SY, Van Treuren T, Nguyen LH (2016). Hepatic Arterial Infusion of Low-Density Lipoprotein Docosahexaenoic Acid Nanoparticles Selectively Disrupts Redox Balance in Hepatoma Cells and Reduces Growth of Orthotopic Liver Tumors in Rats. Gastroenterology.

[135] Zhao C, Cao W, Zheng H, Xiao Z, Hu J, Yang L (2019). Acid-responsive nanoparticles as a novel oxidative stress-inducing anticancer therapeutic agent for colon cancer. Int J Nanomedicine.

[136] de Carvalho TG, Garcia VB, de Araujo AA, da Silva Gasparotto LH, Silva H, Guerra GCB (2018). Spherical neutral gold nanoparticles improve anti-inflammatory response, oxidative stress and fibrosis in alcohol-methamphetamine-induced liver injury in rats. Int J Pharm.

[137] Chen G, Deng H, Song X, Lu M, Zhao L, Xia S (2017). Reactive oxygen species-responsive polymeric nanoparticles for alleviating sepsis-induced acute liver injury in mice. Biomaterials.

[138] Bertrand N, Wu J, Xu X, Kamaly N, Farokhzad OC (2014). Cancer nanotechnology: the impact of passive and active targeting in the era of modern cancer biology. Adv Drug Deliv Rev.

[139] Maeda H, Nakamura H, Fang J (2013). The EPR effect for macromolecular drug delivery to solid tumors: Improvement of tumor uptake, lowering of systemic toxicity, and distinct tumor imaging *in vivo*. Adv Drug Deliv Rev.

[140] Kamaly N, Xiao Z, Valencia PM, Radovic-Moreno AF, Farokhzad OC (2012). Targeted polymeric therapeutic nanoparticles: design, development and clinical translation. Chem Soc Rev.

[141] Liu D, Bimbo LM, Makila E, Villanova F, Kaasalainen M, Herranz-Blanco B (2013). Co-delivery of a hydrophobic small molecule and a hydrophilic peptide by porous silicon nanoparticles. J Control Release.

[142] Moghimi SM, Hunter AC (2001). Capture of stealth nanoparticles by the body's defences. Crit Rev Ther Drug Carrier Syst.

[143] Chen ZG (2010). Small-molecule delivery by nanoparticles for anticancer therapy. Trends Mol Med.

[144] Harris JM, Chess RB (2003). Effect of pegylation on pharmaceuticals. Nat Rev Drug Discov.

[145] Gabizon A, Martin F (1997). Polyethylene glycol-coated (pegylated) liposomal doxorubicin. Rationale for use in solid tumours. Drugs.

[146] Perry JL, Reuter KG, Kai MP, Herlihy KP, Jones SW, Luft JC (2012). PEGylated PRINT nanoparticles: the impact of PEG density on protein binding, macrophage association, biodistribution, and pharmacokinetics. Nano Lett.

[147] Seymour LW, Ferry DR, Anderson D, Hesslewood S, Julyan PJ, Poyner R (2002). Hepatic drug targeting: phase I evaluation of polymer-bound doxorubicin. J Clin Oncol.

[148] Bartlett DW, Su H, Hildebrandt IJ, Weber WA, Davis ME (2007). Impact of tumor-specific targeting on the biodistribution and efficacy of siRNA nanoparticles measured by multimodality *in vivo* imaging. Proc Natl Acad Sci U S A.

[149] Totsuka M, Watanabe Y, Asai C, Takahashi S, Ishikawa H, Takamura N (2019). Case of severe bullous erythema including intertrigo-like eruptions with angioedema induced by pegylated liposomal doxorubicin. J Dermatol.

[150] Ashfaq UA, Riaz M, Yasmeen E, Yousaf MZ (2017). Recent Advances in Nanoparticle-Based Targeted Drug-Delivery Systems Against Cancer and Role of Tumor Microenvironment. Crit Rev Ther Drug Carrier Syst.

[151] Lim WT, Tan EH, Toh CK, Hee SW, Leong SS, Ang PC (2010). Phase I pharmacokinetic study of a weekly liposomal paclitaxel formulation (Genexol-PM) in patients with solid tumors. Ann Oncol.

[152] Chen B, Dai W, He B, Zhang H, Wang X, Wang Y (2017). Current Multistage Drug Delivery Systems Based on the Tumor Microenvironment. Theranostics.

[153] Xu X, Wu J, Liu Y, Saw PE, Tao W, Yu M (2017). Multifunctional Envelope-Type siRNA Delivery Nanoparticle Platform for Prostate Cancer Therapy. ACS Nano.

[154] Kim JY, Lee DY, Kang S, Miao W, Kim H, Lee Y (2017). Bilirubin nanoparticle preconditioning protects against hepatic ischemia-reperfusion injury. Biomaterials.

[155] Fukumura D, Jain RK (2007). Tumor microvasculature and microenvironment: targets for anti-angiogenesis and normalization. Microvasc Res.

[156] Wang S, Yu G, Wang Z, Jacobson O, Lin LS, Yang W (2019). Enhanced Antitumor Efficacy by a Cascade of Reactive Oxygen Species Generation and Drug Release. Angew Chem Int Ed Engl.

[157] Stylianopoulos T, Poh MZ, Insin N, Bawendi MG, Fukumura D, Munn LL (2010). Diffusion of particles in the extracellular matrix: the effect of repulsive electrostatic interactions. Biophys J.

[158] Uthaman S, Huh KM, Park IK (2018). Tumor microenvironment-responsive nanoparticles for cancer theragnostic applications. Biomater Res.

[159] Yang Z, Dai Y, Yin C, Fan Q, Zhang W, Song J (2018). Activatable Semiconducting Theranostics: Simultaneous Generation and Ratiometric Photoacoustic Imaging of Reactive Oxygen Species *In vivo*. Adv Mater.

[160] Johnson RP, Uthaman S, John JV, Lee HR, Lee SJ, Park H (2015). Poly(PEGA)-b-poly(L-lysine)-b-poly(L-histidine) Hybrid Vesicles for Tumoral pH-Triggered Intracellular Delivery of Doxorubicin Hydrochloride. ACS Appl Mater Interfaces.

[161] Zheng D, Li B, Xu L, Zhang QL, Fan JX, Li CX (2018). Normalizing Tumor Microenvironment Based on Photosynthetic Abiotic/Biotic Nanoparticles. ACS Nano.

[162] Wang S, Wang Z, Yu G, Zhou Z, Jacobson O, Liu Y (2019). Tumor-Specific Drug Release and Reactive Oxygen Species Generation for Cancer Chemo/Chemodynamic Combination Therapy. Adv Sci (Weinh).

[163] Liang H, Zhou Z, Luo R, Sang M, Liu B, Sun M (2018). Tumor-specific activated photodynamic therapy with an oxidation-regulated strategy for enhancing anti-tumor efficacy. Theranostics.

[164] Watal G, Watal A, Rai PK, Rai DK, Sharma G, Sharma B (2013). Biomedical applications of nano-antioxidant. Methods Mol Biol.

[165] Sharma A, Madhunapantula SV, Robertson GP (2012). Toxicological considerations when creating nanoparticle-based drugs and drug delivery systems. Expert Opin Drug Metab Toxicol.

[166] Saab AM, Tundis R, Loizzo MR, Lampronti I, Borgatti M, Gambari R (2012). Antioxidant and antiproliferative activity of Laurus nobilis L. (Lauraceae) leaves and seeds essential oils against K562 human chronic myelogenous leukaemia cells. Nat Prod Res.

[167] Aggarwal BB, Bhardwaj A, Aggarwal RS, Seeram NP, Shishodia S, Takada Y (2004). Role of resveratrol in prevention and therapy of cancer: preclinical and clinical studies. Anticancer Res.

[168] Lacatusu I, Badea N, Badea G, Oprea O, Mihaila MA, Kaya DA (2015). Lipid nanocarriers based on natural oils with high activity against oxygen free radicals and tumor cell proliferation. Materials Science and Engineering: C.

[169] Mata R, Nakkala JR, Sadras SR (2015). Biogenic silver nanoparticles from Abutilon indicum: their antioxidant, antibacterial and cytotoxic effects *in vitro*. Colloids Surf B Biointerfaces.

[170] Shahriary M, Veisi H, Hekmati M, Hemmati S (2018). *In situ* green synthesis of Ag nanoparticles on herbal tea extract (Stachys lavandulifolia)-modified magnetic iron oxide nanoparticles as antibacterial agent and their 4-nitrophenol catalytic reduction activity. Mater Sci Eng C Mater Biol Appl.

[171] Shi T, Sun X, He Q (2018). Cytotoxicity of Silver Nanoparticles Against Bacteria and Tumor Cells. Current protein & peptide science.

[172] Ovais M, Khalil AT, Raza A, Khan MA, Ahmad I, Islam NU (2016). Green synthesis of silver nanoparticles via plant extracts: beginning a new era in cancer theranostics. Nanomedicine (Lond).

[173] Onitsuka S, Hamada T, Okamura H (2019). Preparation of antimicrobial gold and silver nanoparticles from tea leaf extracts. Colloids Surf B Biointerfaces.

[174] Boomi P, Ganesan RM, Poorani G, Gurumallesh Prabu H, Ravikumar S, Jeyakanthan J (2019). Biological synergy of greener gold nanoparticles by using Coleus aromaticus leaf extract. Mater Sci Eng C Mater Biol Appl.

[175] Bayda S, Hadla M, Palazzolo S, Riello P, Corona G, Toffoli G (2018). Inorganic Nanoparticles for Cancer Therapy: A Transition from Lab to Clinic. Curr Med Chem.

[176] Ajiboye TO, Komolafe YO, Bukoye Oloyede HO, Yakubu MT, Adeoye MD, Abdulsalami IO (2013). Diethylnitrosamine-induced redox imbalance in rat microsomes: protective role of polyphenolic-rich extract from Sorghum bicolor grains. J Basic Clin Physiol Pharmacol.

[177] Noeman SA, Hamooda HE, Baalash AA (2011). Biochemical study of oxidative stress markers in the liver, kidney and heart of high fat diet induced obesity in rats. Diabetol Metab Syndr.

[178] Mohammed M, Abdel-Gawad E, Awwad S, Kandil E, El-Agamy B (2016). Therapeutic role of a synthesized calcium phosphate nanocomposite material on hepatocarcinogenesis in rats. Biochem Cell Biol.

[179] Liu Y, Li P, Lu J, Xiong W, Oger J, Tetzlaff W (2008). Bilirubin possesses powerful immunomodulatory activity and suppresses experimental autoimmune encephalomyelitis. J Immunol.

[180] Lee Y, Kim H, Kang S, Lee J, Park J, Jon S (2016). Bilirubin Nanoparticles as a Nanomedicine for Anti-inflammation Therapy. Angew Chem Int Ed Engl.

[181] Peralta C, Jimenez-Castro MB, Gracia-Sancho J (2013). Hepatic ischemia and reperfusion injury: effects on the liver sinusoidal milieu. J Hepatol.

[182] Zhai Y, Petrowsky H, Hong JC, Busuttil RW, Kupiec-Weglinski JW (2013). Ischaemia-reperfusion injury in liver transplantation-from bench to bedside. Nat Rev Gastroenterol Hepatol.

[183] Li S, Tan HY, Wang N, Zhang ZJ, Lao L, Wong CW (2015). The Role of Oxidative Stress and Antioxidants in Liver Diseases. Int J Mol Sci.

[184] Matough FA, Budin SB, Hamid ZA, Alwahaibi N, Mohamed J (2012). The role of oxidative stress and antioxidants in diabetic complications. Sultan Qaboos Univ Med J.

[185] Zhu X, Lei X, Wang J, Dong W (2019). Protective effects of resveratrol on hyperoxia-induced lung injury in neonatal rats by alleviating apoptosis and ROS production.

[186] Yan B, Cheng L, Jiang Z, Chen K, Zhou C, Sun L (2018). Resveratrol Inhibits ROS-Promoted Activation and Glycolysis of Pancreatic Stellate Cells via Suppression of miR-21. Oxid Med Cell Longev.

[187] Subedi L, Lee TH, Wahedi HM, Baek SH, Kim SY (2017). Resveratrol-Enriched Rice Attenuates UVB-ROS-Induced Skin Aging via Downregulation of Inflammatory Cascades. Oxid Med Cell Longev.

[188] Yuan Y, Xue X, Guo RB, Sun XL, Hu G (2012). Resveratrol enhances the antitumor effects of temozolomide in glioblastoma via ROS-dependent AMPK-TSC-mTOR signaling pathway. CNS Neurosci Ther.

[189] Mahgoub E, Kumaraswamy SM, Kader KH, Venkataraman B, Ojha S, Adeghate E (2017). Genipin attenuates cisplatin-induced nephrotoxicity by counteracting oxidative stress, inflammation, and apoptosis. Biomed Pharmacother.

[190] Yao ML, Gu J, Zhang YC, Wang N, Zhu ZH, Yang QT (2015). [Inhibitory effect of Genipin on uncoupling protein-2 and energy metabolism of androgen-independent prostate cancer cells]. Zhonghua Nan Ke Xue.

[191] Liu YG, Dai QL, Wang SB, Deng QJ, Wu WG, Chen AZ (2015). Preparation and *in vitro* antitumor effects of cytosine arabinoside-loaded genipin-poly-l-glutamic acid-modified bacterial magnetosomes. Int J Nanomedicine.

[192] Deep G, Singh RP, Agarwal C, Kroll DJ, Agarwal R (2006). Silymarin and silibinin cause G1 and G2-M cell cycle arrest via distinct circuitries in human prostate cancer PC3 cells: a comparison of flavanone silibinin with flavanolignan mixture silymarin. Oncogene.

[193] Wang Y, Zhang L, Wang Q, Zhang D (2014). Recent advances in the nanotechnology-based drug delivery of Silybin. J Biomed Nanotechnol.

[194] Chidambara Murthy KN, Jayaprakasha GK, Singh RP (2002). Studies on antioxidant activity of pomegranate (Punica granatum) peel extract using *in vivo* models. J Agric Food Chem.

[195] Kaur G, Jabbar Z, Athar M, Alam MS (2006). Punica granatum (pomegranate) flower extract possesses potent antioxidant activity and abrogates Fe-NTA induced hepatotoxicity in mice. Food Chem Toxicol.

